# Structure-Guided Prioritization and Synthesis of New Ligands for GPR17 Receptor

**DOI:** 10.1021/acsomega.6c00828

**Published:** 2026-06-06

**Authors:** Marco Rabuffetti, Francesca Rinaldi, Luca Palazzolo, Davide Bianchi, Maria Letizia Trincavelli, Irene Balloni, Simona Daniele, Stefano Capaldi, Giovanna Speranza, Ivano Eberini, Enrica Calleri

**Affiliations:** † Dipartimento di Chimica, 9304 Università degli Studi di Milano, via Golgi 19, Milano 20133, Italy; ‡ Dipartimento di Scienze del Farmaco, Università di Pavia, via Taramelli 12, Pavia 27100, Italy; § Dipartimento di Scienze Farmacologiche e Biomolecolari “Rodolfo Paoletti”, Università degli Studi di Milano, via Giuseppe Balzaretti 9, Milano 20133, Italy; ∥ Dipartimento di Farmacia, Università di Pisa, via Bonanno 6, Pisa 56126, Italy; ⊥ Dipartimento di Biotecnologie, 9310 Università di Verona, Strada Le Grazie 15, Verona 37134, Italy

## Abstract

GPR17, a G protein-coupled receptor (GPCR) involved in neurodegenerative and repair processes, represents a promising therapeutic target. In this study, an integrated strategy combining structure-guided prioritization of a focused nucleotide-inspired library with experimental assays was employed to identify new GPR17 antagonists. A library of 130 nucleosides, nucleotides, and related derivatives was prioritized by molecular docking, leading to the selection of four candidates (8-methylamino inosinic acid and *N* ^2^-*n*-octyl-, *N* ^2^-butyryl- and *N* ^2^-undecanoyl-2′,3′-*O*-isopropylideneguanylic acid). These compounds were synthesized and evaluated using Grating-Coupled Interferometry (GCI), which confirmed nanomolar affinities comparable to the reference antagonist Cangrelor. Functional [^35^S]­GTPγS binding assays demonstrated that all tested compounds act as GPR17 antagonists, with *N* ^2^-*n*-octyl-, *N* ^2^-butyryl- and *N* ^2^-undecanoyl-2′,3′-*O*-isopropylideneguanylic acids showing the highest potency. This integrated approach, combining computational modeling, targeted synthesis, and experimental validation, provides a solid foundation for the rational design of selective GPR17 ligands and paves the way for future optimization efforts aimed at therapeutic applications in neurodegenerative disorders and neural tissue repair.

## Graphical abstract

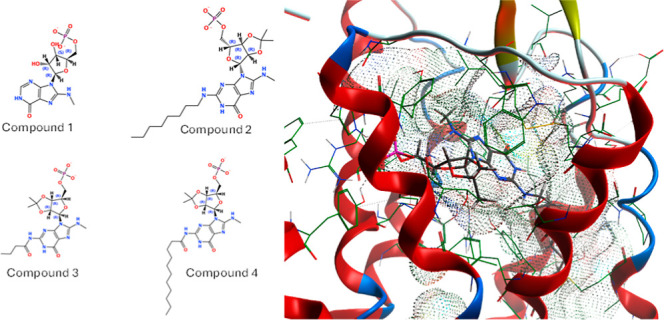

## 1 Introduction

G protein-coupled receptors (GPCRs) are among the most diverse group of membrane receptors present in eukaryotic organisms. Owing to their crucial role in signal transmission from the extracellular environment to the cell interior, they are of great relevance to drug development processes.^1–3^

Nevertheless, among the GPCRs considered druggable, only a small proportion (25–40%) are currently being targeted. The reason is that they are transmembrane receptors and, as such, their investigation presents several challenges.^4,5^ Given these premises, GPCR research is highly significant in medicinal chemistry and pharmacology, with considerable energies dedicated to discovering new ligands able to operate them.

Among all the GPCR receptors, GPR17, mainly expressed in the brain, heart, and kidneys,^6,7^ has been under investigation in the recent past. Considered orphan for many years, since the late 2000s research efforts have been especially focused on studying its involvement in processes such as neuronal myelination.^6–10^ Medical observations confirm that ischemic, traumatic, or demyelinating disturbances are indeed characterized by up-regulation of GPR17 in the central nervous system (CNS),^11,12^ leading to an enhancement of tissue regeneration via cell remyelination in the early steps of lesion development.^13^ High expression of GPR17 is also observed in the necrotic core following spinal cord trauma.^14^ Therefore, all the factors listed above suggest that pharmacological treatment with either GPR17 agonists or antagonists may reduce the extent of neurodegenerative damage.^15^

The GPR17 receptor has been found to be responsive to agonists such as nucleotides and their adducts (UDP, UDP-glucose), as well as radically different messengers (e.g., cysteinyl leukotrienes (LTE4, LTD4 and LTC4), oxysterols and a wide variety of molecules having quite diverse structures).^7^ Other examples of agonist agents are Asinex 1 ^16^ and Galinex,^17^ both bearing a phenyltriazole core. Among the antagonists, the antiplatelet drug Cangrelor (Kengreal or Kengrexal)^18^ and the antiasthmatic Montelukast (Singulair)^19^ are worth mentioning (Figure 1).

**Figure 1:**
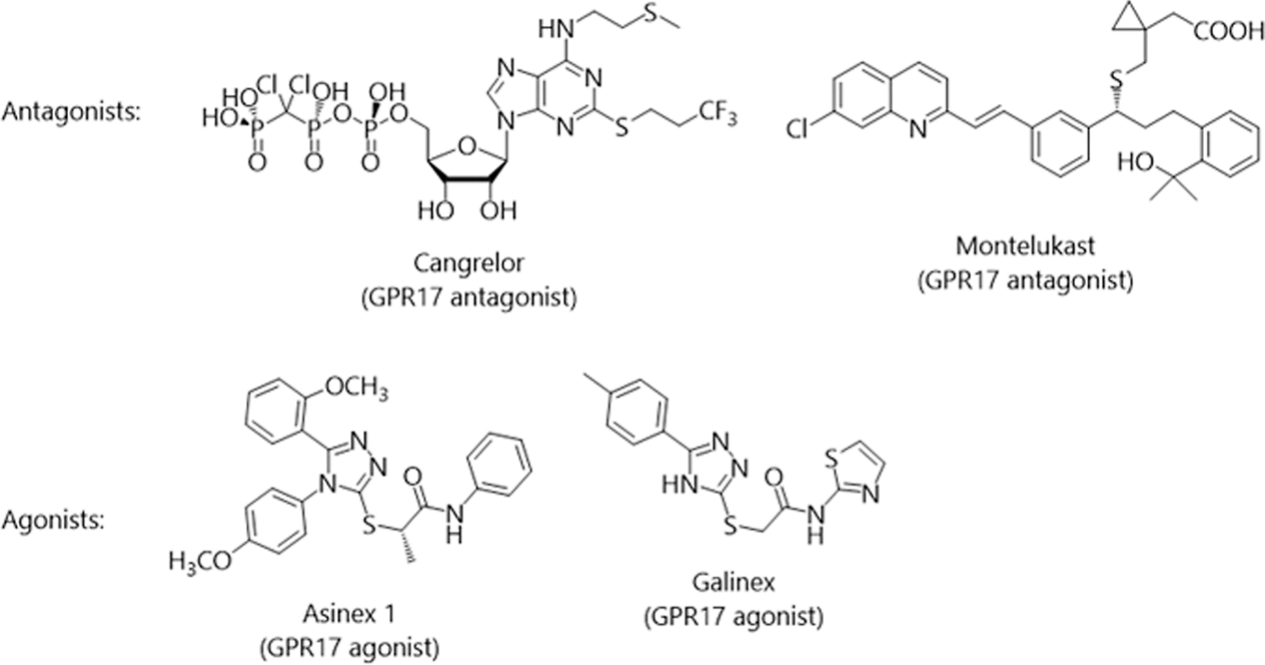
Structures of Cangrelor and Montelukast (GPR17 antagonists), Asinex 1 and Galinex (GPR17 agonists).

Moreover, GPR17 shares structural and functional features with the chemokine receptors CXCR2 and CXCR4, mutually influencing their activity through direct interactions.^20,21^

Structure-guided computational prioritization can support ligand discovery by ranking compounds from chemically focused libraries through molecular docking and related scoring procedures. In this work, a focused in-house library of 130 nucleosides, nucleotides, related analogues, and synthetic intermediates, all deriving from previous synthetic campaigns carried out in our laboratories,^22–26^ was selected for structure-guided computational prioritization. This choice was primarily aided by the existence of well-known GPR17 inhibitors with nucleotide-like structures, such as Cangrelor.

To construct a first 3D structure of GPR17, we employed a homology modeling approach due to the absence of an experimentally determined structure at the time we performed the first part of the research. With the recent publication of the GPR17 crystal structure in its self-activated apo conformation (PDB ID: 7Y89),^27^ the model was refined accordingly for the molecular docking-based prioritization.

The above-mentioned library was then subjected to a computational prioritization workflow. Among the compounds with the best potential to bind to the target, 8-methylaminoinosinic acid (**1**) and three *N* ^2^-alkyl/acyl derivatives of guanylic acid (**2–4**) were selected and synthesized for subsequent binding assays (Figure 2). To the best of our knowledge, neither of these compounds has ever been synthesized and/or characterized to date, despite their dephosphorylated precursors having been reported as intermediates in the preparation of nucleotides.^26^

**Figure 2:**
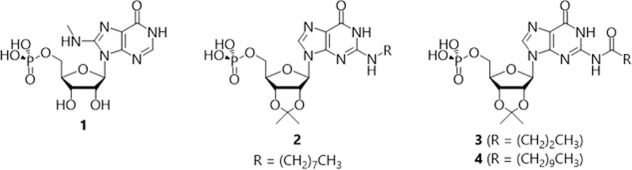
Synthesized compounds for GPR17 binding assays.

The synthesized compounds were considered for the experimental assessment by grating-coupled interferometry (GCI), an evolution of surface plasmon resonance (SPR) based on the same optical principles.

The experimental assessment also included the evaluation of the functional activity of the studied compounds by a reference functional assay ([^35^S]­GTPγS binding assay) to establish their agonist or antagonist properties.

## 2 Results and Discussion

### 2.1 Choice of Molecules for In Silico Computations (Design and Preparation of a Chemical Library)

A focused in-house library comprising nucleosides, nucleotides, related analogues, and synthetic intermediates from previous work carried out in our laboratories^22–26^ was selected for a preliminary in silico prioritization campaign, for a total of 130 compounds. The collection represented a chemically focused, congeneric nucleotide-inspired set rather than a broad exploration of unrelated scaffolds.

This choice was aided by (1) the existence of well-known GPR17 inhibitors with nucleotide-like structures, such as Cangrelor; (2) the activities already exhibited by some of the selected compounds (e.g., as food enhancers, *umami* taste-eliciting molecules and substrates/inhibitors of purine nucleoside phosphorylases (PNPs). All synthetic intermediates, both isolated and not, were also added to the pool to widen the preliminary investigation as much as possible.

### 2.2 In Silico Computations

To identify novel GPR17 ligands, we carried out a structure-guided prioritization of a focused in-house library comprising 130 nucleoside-, nucleotide-, and related analogues. An initial docking-based prioritization was performed with molecular operating environment (MOE) using our previously developed GPR17 homology model based on the inactive P2Y1 receptor structure (PDB ID: 4XNW); after publication of the GPR17 structure (PDB ID: 7Y89), the selected compounds were redocked in the refined hybrid GPR17 model to maintain consistency between the preliminary screening and the final binding-mode analysis. The top-ranked compounds from the first-pass prioritization were then redocked into this refined hybrid model, and the final docking scores and ranking reported in Table 1 refer to this refined receptor structure.

**Table 1:** GBVI/WSA dG and Rescoring Value (LigX) for the Top Scoring Chemicals^a^

name	docking score (GBVI/WSA dG)	rescoring value (LigX)
**4**	–10.4	–12.9
**2**	–10.2	–10.5
cangrelor	–10.1	–11.1
**3**	–9.14	–10.0
asinex1	–8.73	–8.74
**1**	–7.54	–8.19

a All the values are expressed in kcal/mol. Here, the scores obtained with the refined hybrid GPR17 model are reported, whereas the original 4XNW-based homology model was used only for the initial library prioritization.

Table 1 presents the docking scores (GBVI/WSA dG) and refined binding affinities (LigX).

The top scoring chemical structures are reported in the Supporting Information (Figure S1), as well as their binding mode (Figures S2 and S3). Docking-pose inspection revealed a recurrent recognition pattern across the ligand series. The phosphate-containing moiety was generally oriented toward a polar region in the upper part of the GPR17 pocket, where Gln211 emerged as the most recurrent interaction partner and was frequently accompanied by Asn307; additional ligand-dependent contributions involved Arg115, Arg308, Gln199, and Ser218. In contrast, the nucleobase/heteroaromatic portion of the ligands occupied a deeper region of the cavity surrounded by Tyr140, Phe139, Cys132, Gly136, Leu194, Leu198, and Leu210, suggesting that this portion of the scaffold mainly contributes to shape complementarity and local packing.

Cangrelor displayed the most extended interaction network, consistent with its highly functionalized nucleotide-like structure. Its phosphate groups engaged the upper polar region of the pocket, while the central heterocyclic scaffold remained properly accommodated in the inner cavity. The tested analogues preserved this general binding logic but sampled only subsets of the interaction pattern, which may explain their weaker or more heterogeneous affinities.

The docking poses also provide a plausible rationale for the observed SAR trend associated with substituent length. In particular, the lower affinity of compound 4 compared with compound 3 is consistent with steric limitations in the peripheral lipophilic region of the binding site. In the model, this region appears to be delimited by Leu194, Leu225, Met191, Ala190, and Pro198, suggesting that elongation of the substituent is not fully compensated by additional favorable contacts and may instead perturb the optimal positioning of the nucleotide core and its phosphate anchoring interactions.

As already stated, none of the four selected compounds has ever been reported in the literature. In particular, the chemical synthesis of **2**, **3** and **4** can prove challenging due to the acid-labile character of *O*-isopropylidenes, which decompose in the usually strongly acidic conditions of chemical phosphorylation protocols.^26^

### 2.3 Synthesis of Inosinic and Guanylic Acids

Target compounds **1–4** were synthesized according to Scheme 1 (for a more comprehensive version, see Supporting Information, paragraph S2).

**Scheme 1:**
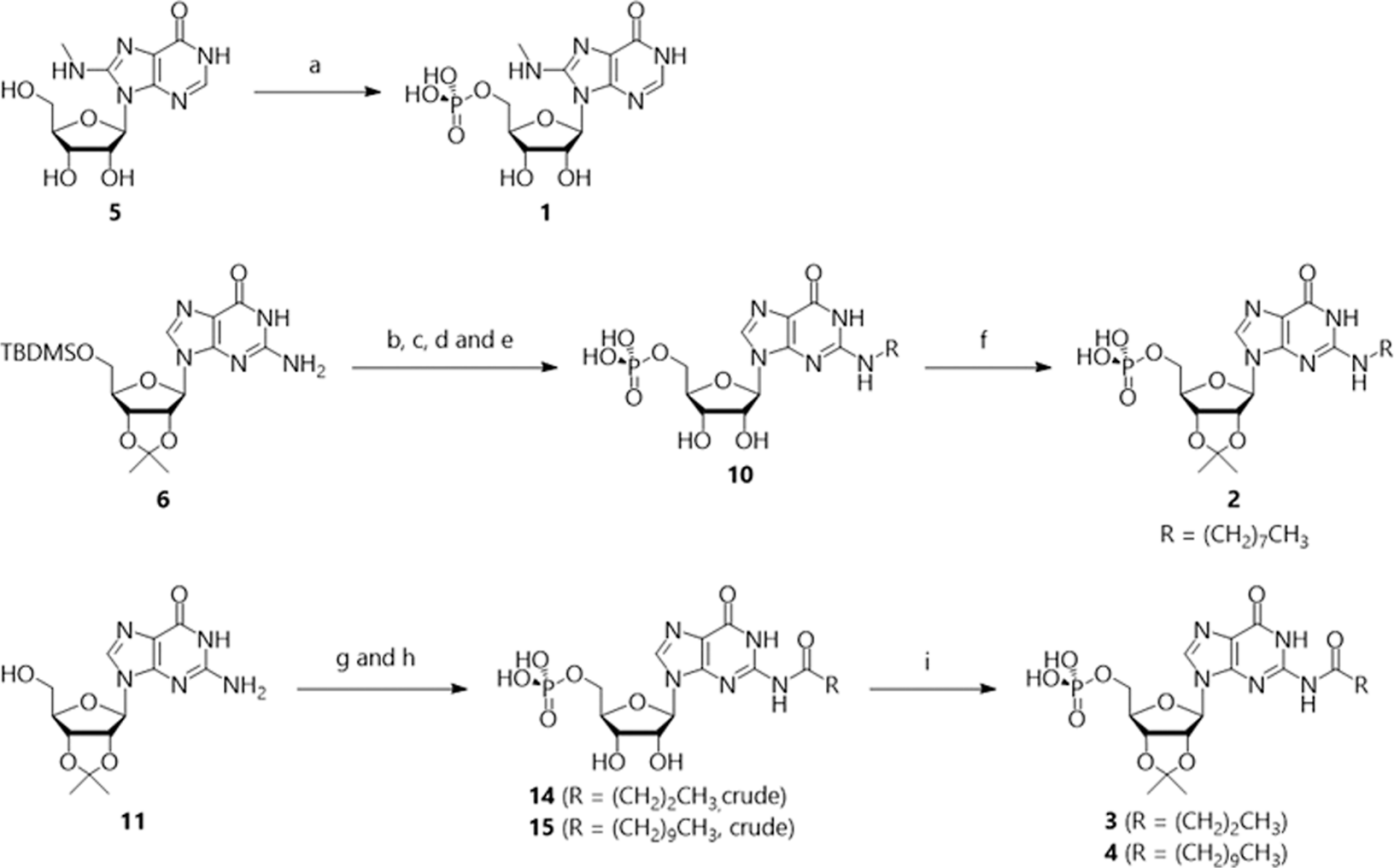
Synthetic Strategy for the Preparation of Target Compounds **1**, **2**, **3** and **4** ^a^

a Reagents and Conditions: (a,e,h): (i) triethylphosphate, 50 °C; (ii) POCl_3_, H_2_O, 0 °C; (iii) H_2_O, 6 M NaOH (to pH 2); (iv) 70 °C (76% for **1**, 73% for **10**, n.d. for **14** and **15**); (b) TMSBr, *t*-BuONO, CH_2_Br_2_, −10 °C → 5-10 °C (69%); (c) *n*-Octylamine, EtOH, MW, 110 °C, 300 W, 6 h (93%); (d) TBAF·3H_2_O, THF (60%); (f,i): 2,2-dimethoxypropane, *p*-toluenesulfonic acid monohydrate, acetone/DMF, reflux (81% for **2**, 30% for **3** (calculated from **12**) and 79% for **4** (calculated from **13**)); (g): (i) TMSCl, pyridine, CH_2_Cl_2_; (ii) RCOX (X = OCO­(CH_2_)_2_CH_3_ for **12** and Cl for **13**); (iii) 1 M HCl, THF (63% for **12** and 62% for **13**).

The synthesis of 8-methylaminoinosinic acid (**1**) was achieved by direct phosphorylation of the 5′–OH moiety of 8-methylaminoinosine (**5**), which was carried out with excess POCl_3_ as phosphorylating agent and triethylphosphate as solvent, according to literature.^28^

As for *N* ^2^-*n*-octyl-2′,3′-*O*-isopropylideneguanylic acid (**2**), position 2 of the purine ring in 2′,3′-*O*-isopropylidene-5′-*O*-*t*-butyldimethylsilylguanosine (**6**) was activated by introducing a bromine leaving group to be displaced by *n*-octylamine under MW irradiation. To perform the bromination step, *tert*-butylnitrite and CH_2_Br_2_ were employed in carefully controlled and previously optimized conditions.^26^ Phosphorylation with POCl_3_ was carried out after desilylation of the 5′–OH group. Since the 2′,3′-*O*-isopropilydene moiety could not be preserved in the strongly acidic phosphorylating conditions, it was reintroduced with 2,2-dimethoxypropane and *p*-toluenesulfonic acid to get product **2**.

To obtain the final *N* ^2^-acyl-2′,3′-*O*-isopropylideneguanylic acids **3** and **4**, the exocyclic amino group of 2′,3′-*O*-isopropylideneguanosine (**11**) was functionalized with the proper acylating agent after temporary protection of the guanine ring with excess chlorotrimethylsilane and pyridine, according to a previously exploited synthetic route.^22^ Similarly to **2**, phosphorylation with POCl_3_ and treatment with 2,2-dimethoxypropane and *p*-toluenesulfonic acid afforded *N* ^2^-butyryl- and *N* ^2^-undecanoyl-2′,3′-*O*-isopropylideneguanylic acids (**3** and **4**, respectively).

### 2.4 GCI Affinity Measurement of the Selected Compounds

Grating-Coupled Interferometry (GCI) was applied to define the affinity of the selected molecules for GPR17. GCI consists in an evolution of SPR, the gold standard in the measurement of biomolecular interactions.

The use of SPR and GCI to study GPCRs is still very challenging. The main issue is related to the fact that GPCRs are membrane proteins and therefore they are not stable outside their natural lipid environment, requiring the creation of a membrane-like environment inside the instrument.^29^

Starting from a previous work of our research group,^16^ a new protocol for GPR17 was setup based on two strategies to stabilize the receptor outside the membranes, namely the use of an engineered GPR17 with stabilizing mutations and the application of an appropriate protocol including stabilizing detergents for the extraction of the receptor from membranes. This procedure allowed to capture GPR17 on the GCI chip directly from the detergent-solubilized membrane extracts avoiding purification, another potential source of instability.

Therefore, the analytical transfer of an SPR method previously developed on a Pioneer AE instrument^16^ was performed on a Creoptix WAVE GCI system. In fact, most GPCR ligands are small molecules and their low molecular weight makes them difficult to detect by SPR. GCI is characterized by higher sensitivity and signal-to-noise ratio compared to SPR and it is considered one of the most sensitive techniques among label-free biosensors.

Before capturing the GPR17 receptor on the sensor chip for GCI analyses, it was necessary to extract it from crude membranes. In an earlier stage of the project,^16^ a suitable protocol was developed allowing to extract engineered GPR17 from crude membranes, immobilize it on a sensor chip and maintain its stability and binding activity for over 24 h after immobilization. The same procedure was applied in this work and includes the use of an appropriate solubilization buffer containing a detergent mix for the effective extraction of the receptor from the isolated membranes, as described in the section Materials and methods.

After incubation and centrifugation of the solution, the supernatant was used for GCI analyses and the receptor was captured directly from the detergent solubilized membrane extracts, avoiding the costly and time-consuming purification.

In brief, crude cell supernatant was therefore injected across a linear polycarboxylate (4PCH) sensor chip in the GCI system where an anti-His_6_ antibody was previously immobilized by amine coupling. In fact, the engineered receptor presents a 10xHis-tag which allows the capture by the anti-His_6_ antibody, ensuring the correct orientation of the receptor for the interaction with its ligands.

The effectiveness of the extraction and capturing procedure was assessed by kinetic analyses of two known GPR17 interactors: the high affinity antagonist Cangrelor and agonist Asinex 1. The affinity order resulted in agreement with results using the Pioneer SPR,^16^ with Asinex 1 showing a lower dissociation constant and a consequent higher affinity compared to Cangrelor. These results confirmed the successful transfer of the SPR method to the Creoptix WAVE GCI system.

Then, the ribonucleotides identified by docking studies were analyzed by GCI applying the same method used for Cangrelor and Asinex 1. Results of the kinetic analyses showed that the docking-prioritized compounds directly interacted with GPR17 and, in most cases, displayed affinities equal to or higher than that of Cangrelor (Table 2 and Figures S16–S21). These data support the qualitative usefulness of our computational workflow, which should be interpreted as a structure-guided prioritization rather than a quantitative affinity prediction. Within this framework, the docking-selected compounds were experimentally confirmed to bind GPR17, and most of them displayed affinities comparable to or higher than Cangrelor, supporting the usefulness of the approach as a prospective enrichment strategy.

**Table 2:** Results of the GCI Kinetic Analyses

molecule	*K*_a_ (M^–1^ s^–1^)	*K*_d_ (s^–1^)	*K*_d_ (nM)
cangrelor	1.42 × 10^5^	1.13 × 10^–1^	792
asinex 1	7.59 × 10^5^	1.25 × 10^–1^	164
**1**	5.15 × 10^5^	8.61 × 10^–2^	167
**2**	3.93 × 10^5^	1.20 × 10^–1^	306
**3**	3.16 × 10^5^	1.28 × 10^–1^	406
**4**	4.53 × 10^4^	3.82 × 10^–2^	843

### 2.5 [^35^S] GTPγS Binding Assay

To further validate the ability of the compounds to interact with GPR17, we tested the molecules in the [^35^S]­GTPγS binding assay, a well-established pharmacological assay suitable for studying GPCR activity.^7,30^ When the compounds were tested alone, no significant stimulation of GTPγS binding to G protein was evidenced (data not shown), thus suggesting that the compounds are not agonists of human GPR17.

In contrast, when increasing concentrations of the compounds were tested in the presence of the GPR17 agonist UDP-glucose (UDP-g), all the molecules were able to decrease the UDP-g-elicited stimulation of GTPγS binding. The effects were evidenced in a concentration-dependent manner, and the compounds displayed IC_50_ values still in the middle–high nanomolar range (Figure 3), thus demonstrating their antagonistic profile to human GPR17 and a good correspondence with the binding data. In particular, among the tested derivatives, **2** and **3** exhibited the highest ability to counteract UDP-g-stimulated binding, with IC_50_ values around 200 nM.

**Figure 3:**
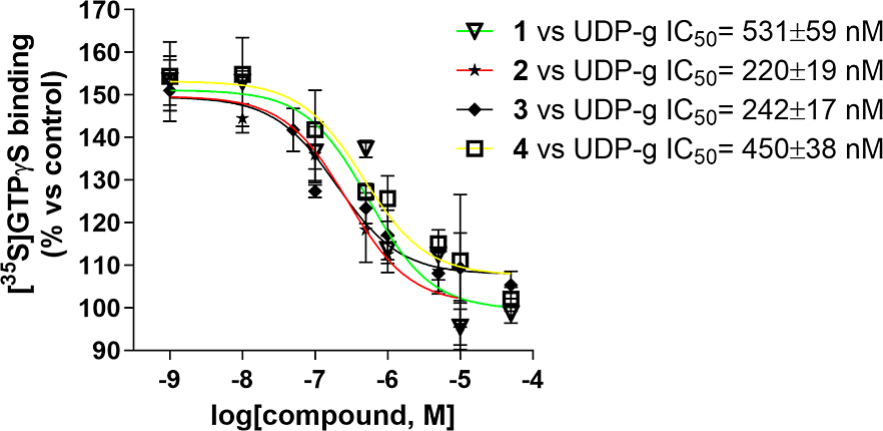
Dose–response curves of GPR17 ligands by the [^35^S]­GTPγS binding assay. Membrane aliquots obtained from 1321N1 cells transfected with HA-tag GPR17 were incubated with UDP-g (50 μM), in the absence or in the presence of different compounds concentrations, and [^35^S]­GTPγS binding assay was performed as described in the Methods section. The data are expressed as percentage of basal [^35^S]­GTPγS binding (set to 100%) and are mean ± SEM of 3 different experiments, each one performed in duplicate.

Taken together, our binding and functional data, considered in the context of previously published biophysical and modeling studies, are most consistent with an orthosteric, purinergic mode of interaction.^16,31^

On this basis, we propose that the compounds characterized here most plausibly act as competitive antagonists at the purinergic orthosteric site, thereby inhibiting UDP glucose–stimulated signaling.

Although our functional data clearly support an antagonistic profile, the present results do not yet allow us to fully define the underlying mechanism. In particular, while the observed inhibition of UDP-glucose responses is compatible with a competitive mode of action, this interpretation remains provisional, as no dedicated pharmacological analyses (such as a Schild-type evaluation) were performed. Further studies will therefore be required to conclusively establish the precise nature of the antagonism. Nevetheless, this interpretation reconciles the observed binding kinetics, the functional antagonism in the [^35^S]­GTPγS assay, and the receptor structural models, and provides a clear framework for future structure-based optimization aimed at improving potency and selectivity. In this sense, given the structural similarity between GPR17 and several P2Y family receptors, as well as the nucleotide-like nature of the ligands tested here, potential cross-reactivity with related purinergic receptors cannot be excluded. Previous studies have shown partial overlap in ligand recognition between GPR17 and P2Y receptors, including P2Y_1_, P2Y_12_, and P2Y_14_, particularly for phosphate-containing or uracil-based scaffolds.^27,32–34^ While our study focused on GPR17 and did not include a selectivity panel, future work will be required to assess the affinity and functional activity of these compounds at closely related P2Y receptors.

## 3 Conclusions

In this work, we developed a new strategy based on in silico studies coupled to experimental assays including GCI and [^35^S]­GTPγS binding assay to identify new ligands for GPR17, a receptor that plays a crucial role in the development of ischemic brain damage as well as in the later remodeling/repair processes.

In the present study, a library composed of 130 nucleosides, nucleotides, analogues and intermediates was tested by a structure-guided prioritization, allowing to select 4 potential ligand candidates of GPR17. The selected molecules were synthesized and first analyzed by GCI to determine the kinetics and affinities of the interaction between the compounds and GPR17. Further confirmation was obtained using a functional reference assay ([^35^S]­GTPγS binding assay), which demonstrated that all the 4 molecules are GPR17 antagonists all in the midhigh nanomolar range.

The present study represents the first step toward the integration between GPR17 computational assay and binding and functional analysis for the rational identification of new candidate ligands by selecting only the most promising compounds. While the high polarity of these phosphate-containing molecules currently limits their CNS penetration, they can represent valuable tool antagonists for target validation and provide a new structural perspective for the future design of more lipophilic, drug-like analogues. At this stage, they establish a useful pharmacological basis that paves the way for future medicinal chemistry efforts, such as the use of phosphate bioisosteres or prodrug strategies to enhance their translational utility in neurodegeneration.

## 4 Experimental Section

### 4.1 Materials and Methods

#### 4.1.1 In Silico Computations

All molecules were initially designed in 2D using the Molecular Operating Environment (MOE) software (Version 2022, ChemComp Group, Quebec, Canada) and then imported into a molecular database. They were subsequently converted to 3D configurations. The stereochemistry was carefully examined, and the dominant protomer/tautomer as well as the protonation state were calculated at physiological pH for each chemical. Subsequently, the chemical structures were energy minimized using the AMBER10/EHT force field.

Since our investigation began before the release of the first 3D structure of GPR17, we employed a homology modeling approach due to the absence of an experimentally determined structure at the time we performed the first part of the research. This model was based on a multiple sequence alignment of a structurally related subclass of class-A GPCRs, as previously described in our works.^16,20^ The alignment was conducted using the TM-Coffee algorithm, part of the T-Coffee package optimized for transmembrane proteins.^35^ From this alignment, both P2Y1 and CysLT1 receptors showed high sequence identity with GPR17 (29.6% and 30.3%, respectively), and their phylogenetic proximity to GPR17 made them promising templates. To remain consistent with prior studies,^21^ the X-ray structure of the P2Y1 receptor in complex with the antagonist MRS2500 (PDB ID: 4XNW)^36^ was selected as the template to model GPR17 in its inactive state.

The binding site of GPR17 in its inactive apo conformation was identified using the “Site Finder” tool in MOE, which calculates potential active sites from the receptor 3D atomic coordinates through a geometric approach. This tool generates “dummy atoms” to approximate possible ligand locations within the receptor binding pocket.

To evaluate the ligand binding capabilities, we applied the “Rigid Receptor” docking protocol with an initial Placement phase using the Triangle Matcher method, yielding 10 poses by aligning ligand atom triplets with receptor site points represented as alpha spheres. These poses were scored using the London dG scoring function, which estimates the ligand binding free energy for each pose. The Refinement phase, also in “Rigid Receptor” mode, allowed the ligand to optimize its structure in the binding site while maintaining receptor side chains rigid. The force field-based GBVI/WSA dG scoring function^37^ was used in this phase to evaluate approximately binding free energy, resulting in two final poses that were prioritized for further analyses, including synthesis and in vitro testing. The AMBER10/EHT force field was applied throughout these procedures. This model was used for the preliminary docking-based filtering of the 130-compound library.

After publication of the cryo-EM structure of GPR17 in complex with Gi (PDB ID: 7Y89), the receptor model was refined. The experimental structure was prepared in MOE by removing the Gi protein, adding terminal caps, and adjusting the global charge. Because 7Y89 corresponds to an activated, self-activated GPR17 conformation in which ECL2 occupies the orthosteric cavity, we generated a refined hybrid model by incorporating the solved GPR17 structure while remodeling ECL2 into an open conformation and TM6 into an inactive-like arrangement. The refined model was checked with the MOE Protein Geometry module and compared with the original homology model after superposition of the transmembrane bundle. The resulting RMSD values were 2.2 Å for the overall backbone and 3.0 Å for the binding-pocket residues. The binding pocket comprises 38 residues and, among them, the ECL2 ones weight approximately the 40% of RMSD for the binding pocket. Globally, the hybrid model shows a larger binding pocket due to ECL2 placement. All the TM residues backbone of the binding pocket are perfectly overlapped in the two models confirming the quality of the original P2Y1-derived model.

The top-ranked compounds were subsequently redocked in the refined hybrid model using the same protocol, generating 30 placement poses and 10 refined poses. LigX was also used to estimate affinity and solvation contributions. After docking, LigX-based pocket-ligand minimization was performed to optimize the local interactions within the complex. During this step, amino-acid side chains in the binding pocket were allowed to undergo local rearrangements around the bound ligand, providing limited local adaptation of the orthosteric site.

Globally, the receptor backbone was kept fixed during docking, and no induced-fit docking protocol or molecular dynamics validation was performed for the final ranking. Therefore, docking was used as a qualitative prioritization tool and as a source of binding hypotheses, rather than as a quantitative predictor of absolute affinity. The postdocking LigX minimization allowed only local pocket relaxation, mainly through side-chain adjustment, and should not be interpreted as full conformational sampling of the receptor.

#### 4.1.2 Chemicals and Reagents

All solvents and reagents were purchased from Merck (Darmstadt, Germany) or Avantor (Radnor Township, Pennsylvania, US) and were used without further purification. Solvents for synthesis were distilled before use. All other solvents were of HPLC grade.

Analytical TLC was performed on silica gel F_254_ precoated aluminum sheets (0.2 mm layer, Merck); components were detected under an UV lamp (λ 254 nm) and/or by spraying with a 10% w/v CeSO_4_/(NH_4_)_6_Mo_7_O_24_·4H_2_O solution or a 5% w/v ninhydrin solution in EtOH, followed by heating at 150 °C. Silica gel 60, 40–63 μm (Merck, Darmstadt, Germany), was used for flash column chromatography.

^1^H, ^13^C and ^31^P NMR spectra were recorded at 400.13, 100.61, and 161.98 MHz, respectively, on a *Bruker AVANCE 400* spectrometer equipped with a *TOPSPIN* software package (Bruker, Karlsruhe, Germany) at 300 K, unless stated otherwise. ^1^H and ^13^C chemical shifts (δ) are given in parts per million and were referenced to the solvent signals (δ_H_ 7.26–δ_C_ 77.16, δ_H_ 2.50–δ_C_ 39.52 and δ_H_ 4.79 ppm from tetramethylsilane (TMS) for CDCl_3_, DMSO-*d*_6_ and D_2_O, respectively). ^31^P chemical shifts are referred to 85% H_3_PO_4_ as external standard (δ_P_ 0.00 ppm).

Electrospray ionization mass spectra (ESI-MS) were recorded on a *Thermo Finnigan LCQ Advantage* spectrometer (Hemel Hempstead, Hertfordshire, UK).

Analytical and semipreparative HPLC were performed using an *Amersham Pharmacia Biotech (P900)* chromatographer equipped with a UV–vis detector. Conditions were as follows: column, Jupiter 10 μ Proteo 90A (10 μm, 250 × 4.6 mm (analytical HPLC) and 250 × 10.0 mm (semipreparative HPLC) (Phenomenex, Torrance, California, US); flow rate, 0.5 mL·min^–1^ (analytical HPLC) and 5 mL·min^–1^ (semipreparative HPLC); λ, 226 and 250 nm. Elution methods are reported in each paragraph describing the preparation of the corresponding product. *t*_R_ is always referred to analytical conditions.

For Grating-Coupled Interferometry (GCI) experiments, HEPES, Tween 20, Cangrelor tetrasodium salt, Protease Inhibitor Cocktail, Dodecyl Maltoside (DDM) and Cholesteryl Hemisuccinate (CHS) were from Sigma-Aldrich (Milan, Italy). Sodium chloride (NaCl) and dimethyl sulfoxide (DMSO) were purchased from PanReac AppliChem ITW Reagents (Cinisello Balsamo, Italy). *N*-hydroxysuccinimide (NHS), ethyl-3­(3-dymethylamino) propyl carbodiimide (EDC) and ethanolamine were from XanTec bioanalytics GmbH, Duesseldorf, Germany. The anti-His_6_ antibody and sodium acetate were purchased from Merck KGaA (Darmstadt, Germany). Deionized water was obtained from a Milli-Q Integral purification system from Merck KGaA (Darmstadt, Germany).

### 4.2 Synthesis of Inosinic and Guanylic Acids (1–4)

#### 4.2.1 General 5′-Phosphorylation Procedure

A suspension of the proper substrate (**9**, **12** or **13**) in triethylphosphate (300 μM) was stirred at 50 °C for 15 min. After cooling the solution/suspension to 0 °C, POCl_3_ (6.00 equiv) and H_2_O (1.00 equiv) were added and the resulting mixture was stirred at 0 °C for 6 h. The disappearance of the substrate was monitored by TLC (EtOH–H_2_O, 7:3; *R*_f_ = 0.80–0.90). After diluting with H_2_O (3:1 ratio), the pH was adjusted to 2 with 6 M NaOH and the resulting solution was stirred at 70 °C for 2 h. The mixture was then neutralized with 6 M NaOH, diluted with more H_2_O (20:1 ratio) and freeze-dried. The resulting crude was either used in the next step without further purification or purified by semipreparative HPLC (see Table S1).

#### 4.2.2 8-Methylaminoinosinic Acid (**1**)

The title product was obtained as an off-white powder (24 mg, 64 μmol, 76%), starting from **5** (25 mg, 84 μmol). *R*_f_: 0.67 (EtOH–H_2_O, 7:3). ^1^H NMR (400 MHz, D_2_O): δ (ppm) 8.15 (s, 1H), 6.07 (d, *J* = 7.1 Hz, 1H), 4.68 (br dd, *J* = 7.0, 5.9 Hz, 1H), 4.40 (dd, *J* = 5.6, 2.5 Hz, 1H), 4.33 (br t, *J* = 1.8 Hz, 1H), 4.14 (br dd, *J* = 11.4, 7.0 Hz, 1H), 4.12–4.06 (m, 1H), 3.11 (s, 3H). ^13^C NMR (100 MHz, D_2_O): δ (ppm) 153.2, 148.9, 147.1, 146.8, 111.4, 87.8, 85.0 (d, ^3^ *J*_CP_ = 8.2 Hz), 71.3, 70.1, 64.6 (br d,^2^ *J*_*CP*_ = 3.0 Hz), 29.7. ^31^P NMR (161 MHz, D_2_O): δ (ppm) 0.78. MS (ESI^–^): *m*/*z* calcd for [C_11_H_16_N_5_O_8_P]^−^: 377.07; found, 376.0 [M – H]^−^, 753.0 [2M – H]^−^, 775.1 [2­(M – H)+ Na]^−^.

#### 4.2.3 *N* ^2^-*n*-Octylguanylic Acid (**10**)

The title product was obtained as a white powder (24 mg, 50 μmol, 73%), starting from **9** (30 mg, 69 μmol). *R*_f_: 0.74 (EtOH–H_2_O, 7:3). ^1^H NMR (400 MHz, D_2_O): δ (ppm) 8.00 (s, 1H), 5.91 (d, *J* = 5.6 Hz, 1H), 4.68 (t, *J* = 5.4 Hz, 1H), 4.42 (t, *J* = 4.5 Hz, 1H), 4.22 (br d, *J* = 2.5 Hz, 1H), 4.02 (dd, *J* = 11.7, 4.4 Hz, 1H), 3.97 (dd, *J* = 12.0, 4.6 Hz, 1H), 3.28 (qt, *J* = 3.4, 6.9 Hz, 2H), 1.55–1.45 (m, 2H), 1.30–1.06 (m, 10H), 0.72 (t, *J* = 6.6 Hz, 3H). ^13^C NMR (100 MHz, D_2_O): δ (ppm) 158.0, 152.6, 151.7, 137.0, 114.6, 86.9, 83.5 (d, ^3^ *J*_CP_ = 7.1 Hz), 74.6, 70.2, 64.8 (br d, ^2^ *J*_CP_ = 3.2 Hz), 41.0, 29.0, 31.6, 29.1, 28.8, 26.8, 22.3, 13.6. ^31^P NMR (161 MHz, D_2_O): δ (ppm) 1.44. MS (ESI^–^): *m*/*z* calcd for [C_18_H_30_N_5_O_8_P]^−^: 475.18; found, 474.32 [M – H]^−^, 949.10 [2M-H]^−^, 971.15 [2­(M – H)+ Na]^−^.

#### 4.2.4 *N* ^2^–Butyrylguanylic Acid (**14**)

The title product was obtained as a crude (407 mg) starting from **12** (55 mg, 140 μmol) and used in the next step without further purification. MS (ESI^–^): *m*/*z* calcd for [C_14_H_20_N_5_O_9_P]^−^: 433.10; found, 433.61 [M]^−^, 455.88 [2M + Na]^−^.

#### 4.2.5 *N* ^2^-*n*-Undecanoylguanylic Acid (**15**)

The title product was obtained as crude (464 mg) starting from **13** (69 mg, 140 μmol) and used in the next step without further purification.

#### 4.2.6 General Procedure for the Synthesis 2′,3′-*O*-Isopropylideneguanylic Acids (**2**, **3** and **4**)

2,2-Dimethoxypropane (20.00 equiv) and *p*-toluenesulfonic acid monohydrate (1.0 equiv) were added to a suspension of the proper substrate (**10**) or crude (**14** and **15**) in a dry acetone-DMF mixture (4:1 v/v, 35 μM) under inert atmosphere and the resulting mixture was refluxed for 24 h. As for crudes **14** and **15**, the concentration is referred to the substrate μmoles corresponding to a 100% yield in the previous step. The resulting suspension was neutralized with a saturated NaHCO_3_ solution and freeze-dried.

#### 4.2.7 *N* ^2^-*n*-Octyl-2′,3′-*O*-Isopropilydeneguanylic Acid (**2**)

The crude was purified by semipreparative HPLC to obtain the title product as a white powder (35 mg, 68 μmol, 81%), starting from **10** (40 mg, 84 μmol). *R*_f_: 0.35 (*n*-BuOH-AcOH-H_2_O, 4:1:1). ^1^H NMR (400 MHz, D_2_O): δ (ppm) 8.01 (s, 1H), 6.06 (br d, *J* = 2.0 Hz, 1H), 5.30 (br dd, *J* = 5.0, 2.8 Hz, 1H), 5.05 (br dd, *J* = 6.0, 1.9 Hz, 1H), 4.46 (br dd, *J* = 6.4, 4.1 Hz, 1H), 3.91–3.84 (m, 2H), 3.29 (ddq, *J* = 20.3, 13.6, 6.9 Hz, 2H), 1.57 (s, 3H), 1.56–1.49 (m, 2H), 1.35 (s, 3H), 1.31–1.08 (m, 10H), 0.73 (br t, *J* = 6.7 Hz, 3H). ^13^C NMR (100 MHz, D_2_O): δ (ppm) 158.9, 152.7, 151.4, 137.2, 115.3, 113.8, 89.2, 85.3, 84.4, 81.5, 64.4, 41.1, 31.7, 26.7, 24.7, 29.2, 28.9, 26.9, 22.4, 13.7. ^31^P NMR (161 MHz, D_2_O): δ (ppm) 3.33. MS (ESI^–^): *m*/*z* calcd for [C_21_H_34_N_5_O_8_P]^−^: 515.21; found, 514.24 [M – H]^−^, 1051.07 [2­(M – H)+ Na]^−^.

#### 4.2.8 *N* ^2^–Butyryl-2′,3′-*O*-Isopropylideneguanylic Acid (**3**)

The crude was purified by semipreparative HPLC to obtain the title product as a white powder (16 mg, 34 μmol, 30% calculated from **12**), starting from crude **14** (407 mg). *R*_f_: 0.26 (*n*-BuOH-AcOH-H_2_O, 4:1:1). ^1^H NMR (400 MHz, D_2_O): δ (ppm) 8.16 (s, 1H), 6.10 (d, *J* = 2.8 Hz, 1H), 5.33 (dd, *J* = 6.1, 2.8 Hz, 1H), 5.18 (dd, *J* = 6.1, 2.1 Hz, 1H), 4.55 (dd, *J* = 5.7, 4.4 Hz, 1H), 3.96 (dt, *J* = 11.3, 4.6 Hz, 1H), 3.88 (dd, *J* = 11.3, 5.0 Hz, 1H), 2.47 (t, *J* = 7.4 Hz, 2H), 1.64 (hex, *J* = 7.4 Hz, 2H), 1.58 (s, 3H), 1.37 (s, 3H), 0.90 (t, *J* = 7.4 Hz, 3H). ^13^C NMR (100 MHz, D_2_O): δ (ppm) 178.2, 157.4, 151.7, 147.7, 142.4, 114.6, 90.9, 85.7, 84.1, 81.6, 64.1, 38.4, 26.1, 24.3, 18.1, 12.7. ^31^P NMR (161 MHz, D_2_O): δ (ppm) 2.03. MS (ESI^–^): *m*/*z* calcd for [C_17_H_24_N_5_O_9_P]^−^: 473.13; found, 473.47 [M]^−^, 495.36 [M – H + Na]^−^, 946.56 [2M]^−^, 967.52 [2­(M – H)+Na]^−^, 1418.19 [3M – H]^−^.

#### 4.2.9 *N* ^2^–Undecanoyl-2′,3′-*O*-Isopropylideneguanylic Acid (**4**)

The crude was suspended in H_2_O and the formed precipitate was filtered to obtain the title product as a white powder (65 mg, 114 μmol, 79% calculated from **13**), starting from crude **15** (464 mg). *R*_f_: 0.28 (*n*-BuOH-AcOH-H_2_O, 4:1:1). ^1^H NMR (400 MHz, D_2_O + DMSO-*d*_6_): δ (ppm) 8.02 (s, 1H), 6.18 (d, *J* = 1.6 Hz, 1H), 5.62 (br dd, *J* = 6.4, 2.7 Hz, 1H), 5.13 (dd, *J* = 6.3 Hz, 1H), 4.49 (q, *J* = 10.3 Hz, 1H), 4.34–4.13 (m, 2H), 3.57–3.46 (m, 2H), 2.57 (br t, *J* = 2.6 Hz, 2H), 2.18 (br t, *J* = 7.4 Hz, 2H), 1.51 (s, 3H), 1.34–1.19 (m, 17H), 0.90 (t, *J* = 7.0 Hz, 3H). ^13^C NMR (100 MHz, D_2_O + DMSO-*d*_6_): δ (ppm) 176.6, 155.4, 148.8, 148.0, 147.6, 112.6, 90.9, 85.8, 85.0, 82.3, 62.8, 36.4, 31.8, 29.4, 29.3, 29.1, 29.0, 28.9, 24.9, 22.6, 14.4. ^31^P NMR (161 MHz, D_2_O + DMSO-*d*_6_): δ (ppm) −0.41. MS (ESI^–^): *m*/*z* calcd for [C_24_H_38_N_5_O_9_P]^−^: 571.24; found, 490.28 [M – H-(PO_3_H)]^−^, 570.65 [M – H]^−^, 1141.83 [2M – H]^−^, 1163.79 [2­(M – H)+ Na]^−^.

### 4.3 Expression of GPR17-T4 in Insect Cells and Membrane Preparation

Expression of full-length human GPR17 with T4 lysozyme inserted into ICL3 (GPR17-T4 1–339) in insect cells was performed as described in Capelli et al. 2020 ^16^ with minor modifications. Briefly, suspension cultures of SF9 cells in SF900-III serum-free medium were grown at a density of ∼2 × 10^6^ cells/mL and infected with high titer baculovirus stocks at moi of 3. After 48 h, cells were collected and resuspended in ice-cold Lysis Buffer (10 mM HEPES, pH 7.5, 10 mM MgCl_2_, 20 mM KCl) supplemented with DNase (20 μg/mL) and protease inhibitors. Cells were disrupted with in Dounce homogenizer at 4 °C and the insoluble fraction was collected by ultracentrifugation (45.000 rpm) for 45 min. The pellet was subjected to two washes with lysis buffer, followed by a further wash with high-salt buffer (LB with 1 M NaCl). Subsequently, the membrane fraction was isolated by centrifugation and resuspended in Membrane Storage Buffer (10 mM HEPES, pH 7.5, 10 mM MgCl_2_, 20 mM KCl, 20% glycerol) at a total protein concentration of 3 mg/mL. The resuspended membranes were then divided into small aliquots (200 μL), rapidly frozen in liquid nitrogen, and stored at −80 °C. The expression level of the recombinant protein in different preparations was assessed by Western blot with anti-His-tag primary antibody (Sigma-Merck).

### 4.4 Antibody Immobilization and GPR17 Capturing for GCI Analyses

Grating-Coupled Interferometry (GCI) experiments were carried out on a Creoptix WAVE instrument (Malvern Panalytical, Wädenswil, Switzerland) controlled by a Creoptix WAVEcontrol software (4.3.4 version).

Following a procedure previously described by our research group,^16^ the engineered GPR17 receptor was extracted from crude membranes and captured on a sensor chip by an anti-His_6_ antibody.

Briefly, the anti-His_6_ antibody was immobilized on a 4PCH sensor chip (Creoptix AG, Malvern Panalytical, Wädenswil, Switzerland) by amine coupling, using a running buffer composed of 20 mM HEPES, pH 7.4, 150 mM NaCl and 0.005% Tween 20. Activation of the flow cells was carried out by the injection of a 1:1 mixture of 100 mM *N*-hydroxysuccinimide (NHS) and 400 mM ethyl-3­(3-dymethylamino) propyl carbodiimide (EDC) for 240 s at 10 μL/min, followed by the injection (800 s, 10 μL/min) of the antibody solution (50 μL of a 100 μg/mL antibody solution in water and 300 μL of 10 mM sodium acetate, pH 4.5). For the passivation, 1 M ethanolamine, pH 8.0 was injected for 420 s at a flow rate of 10 μL/min on the chip surface. Approximately 13000 pg/mm^2^ of antibody were immobilized on two channels of the sensor chip.

The antibody was used to capture the engineered 10xHis-tag GPR17 receptor after its extraction from crude membranes. The extraction procedure was carried out as follows: dilution of the crude membrane extract to 5 mg/mL total proteins; addition of Protease Inhibitor Cocktail 100× (5% v/v); incubation on ice for 30 min; dilution in an equal volume of solubilization buffer 2× (100 mM HEPES, pH 7.5, 0.6 M NaCl, 2% Dodecyl Maltoside (DDM)/Cholesteryl Hemisuccinate (CHS) 5:1) and incubation on ice for 2.5 h; centrifugation (40 min, 14000 rpm) and collection of the supernatant.

Crude cell supernatant was then injected across one channel of the sensor chip at a flow rate 10 μL/min for the capture; the second channel was used as a reference. A running buffer composed of 20 mM HEPES, pH 7.4, 150 mM NaCl and 0.03% DDM/CHS 5:1 was used. Three short injections (15 s, 50 μL/min) of sodium chloride in a detergent solution (NaCl 0.5 M in DDM/CHS 5:1) were performed to remove nonspecifically bound supernatant debris. Following this protocol, a level of around 1000 pg/mm^2^ was reached for the capture of GPR17-T4 1–339.

### 4.5 GCI Kinetic Analyses

Kinetic analyses were carried out in a running buffer composed of 20 mM HEPES, pH 7.4, 150 mM NaCl, 0.03% DDM/CHS 5:1 (with 1% DMSO for Asinex 1) and at a constant temperature of 25 °C.

The molecules were tested in serial dilutions, from 15.6 nM to 2 μM for Cangrelor, from 7.8 nM to 1 μM for Asinex 1 and from 125 nM to 2 μM for the compounds identified by docking studies. A dilution factor of 2 was applied and 2 replicates for each concentration were analyzed.

Analytes were injected at a flow rate of 25 μL/min for 60 s on the two channels of the sensor chip. A 240 s dissociation time was found sufficient to guarantee the complete dissociation of the analytes from the receptor. Several buffer blanks were injected for double referencing.

A DMSO calibration (0.5–1.5%) was performed for Asinex 1 to correct for bulk refractive index shifts.

Sensorgrams were processed by the Creoptix WAVEcontrol software (4.5.18 version) using the kinetic analysis evaluation tool, with a double referencing and a 1:1 interaction model.

### 4.6 Cell Culture and Transfection

Astrocitoma cells (1321N1) stably transfected with HA-tag GPR17 ^7,21^ were grown in monolayer and maintained in Dulbecco’s modified Eagle’s medium F12 (DEMEM F12), supplemented with 10% (v/v) FBS, 100 U/ml penicillin, 100 μg/mL streptomycin) at 37 °C with 5% CO_2_ atmosphere until confluent and 1 μg/mL G418. These cells were used for membrane preparation, as reported below.

### 4.7 [^35^S] GTPγS Binding Assay

In order to obtain cell membranes, 1321N1 Hat-tag GPR17 cells were lysed in a glass potter homogenizer with the lysis buffer (5 mM Tris·HCl, 2 mM EDTA, pH 7.4). The lysate was centrifuged at 48,000*g* for 20 min at 4 °C and each pellet was rinsed with wash buffer (50 mM Tris·HCl,10 mM MgCl2, pH 7.4) and homogenized. Proteins were quantified (Bradford assay) and aliquots (500 μg) stored at −80 °C until used.

To determine the functional activity of the new compounds, the [^35^S] GTPgS assay was perfomed. Briefly, 10 μg of membranes from 1321N1-GPR17 cells were diluted in binding buffer (20 mM HEPES, pH 7.4, 100 mM NaCl, 3 mM MgCl_2_) and incubated with increasing concentrations (ranging from 1 nM to 50 μM) of the new compounds, and GTPγS binding to activated G proteins was quantified as previously described.^20,38^ To determine if the new compounds can display a profile of antagonism, the experiments were repeated in the presence of the GPR17 agonist UDP-glucose (50 μM). Samples were incubated for 30 min at 30 °C and the reactions were stopped by rapid filtration under vacuum. The filters were washed four times with binding buffer, removed from the multiplate, and poured in a scintillation cocktail and read at a scintillator counter (TRI-CARB 2900TR, PerkinElmer). For the analysis and graphic presentation of [^35^S]­GTPγS binding data, a nonlinear multipurpose curve fitting computer program (Graph-Pad Prism) was used, from which IC_50_ values were derived. All data are presented as the mean ± SEM of three different experiments.

## Supplementary Material



## Notes

S1. Results of in silico computations ; Figure S1. Top scoring chemical structures derived from in silico computations; Figure S2. 3D van der Waals surface representation of the ligand–receptor complexes for Asinex 1 (A), Cangrelor (B) and compounds 1 (C), 2 (D), 3 (E) and 4 (F); Figure S3. 2D interaction diagrams of ligand binding within the GPR17 binding site for Asinex 1 (A), Cangrelor (B) and compounds **1** (C), **2** (D), **3** (E) and **4** (F); S2. Synthesis of inosinic and guanylic acids (**1**–**4**) and their intermediates; Scheme S1. Synthetic strategy for the preparation of target compounds **1**, **2**, **3** and **4**; S3. HPLC data for the purification of compounds **1**, **2**, **3** and **10**; Table S1. HPLC data for the purification of compounds **1**, **2**, **3** and **10**; S4. ^1^H, ^13^C and ^31^P and NMR spectra of compounds **1**, **2**, **3** and **4**; Figure S4. ^1^H NMR spectra of 8-methylaminoinosinic acid (**1**); Figure S5. ^13^C NMR spectra of 8-methylaminoinosinic acid (**1**); Figure S6. ^31^P NMR spectra of 8-methylaminoinosinic acid (**1**); Figure S7. ^1^H NMR spectra of N^2^-*n*-octyl-2′,3′-*O*-isopropilydeneguanylic acid (**2**); Figure S8. ^13^C NMR spectra of N^2^-*n*-octyl-2′,3′-*O*-isopropilydeneguanylic acid (**2**); Figure S9. ^31^P NMR spectra of N^2^-*n*-octyl-2′,3′-*O*-isopropilydeneguanylic acid (**2**); Figure S10. ^1^H NMR spectra of N^2^-*n*-butyryl-2′,3′-*O*-isopropilydeneguanylic acid (**3**); Figure S11. ^13^C NMR spectra of N^2^-*n*-butyryl-2′,3′-*O*-isopropilydeneguanylic acid (**3**); Figure S12. ^31^P NMR spectra of N^2^-*n*-butyryl-2′,3′-*O*-isopropilydeneguanylic acid (**3**); Figure S13. ^1^H NMR spectra of N^2^-*n*-undecanoyl-2′,3′-*O*-isopropilydeneguanylic acid (**4**); Figure S14. ^13^C NMR spectra of N^2^-*n*-undecanoyl-2′,3′-*O*-isopropilydeneguanylic acid (**4**); Figure S15. ^31^P NMR spectra of N^2^-*n*-undecanoyl-2′,3′-*O*-isopropilydeneguanylic acid (**4**); S5. Results of GCI kinetic analyses; Figure S16. Sensorgrams deriving from the GCI kinetic analysis of Cangrelor; Figure S17. Sensorgrams deriving from the GCI kinetic analysis of Asinex 1; Figure S18. Sensorgrams deriving from the GCI kinetic analysis of **1**; Figure S19. Sensorgrams deriving from the GCI kinetic analysis of **2**; Figure S20. Sensorgrams deriving from the GCI kinetic analysis of **3**; Figure S21. Sensorgrams deriving from the GCI kinetic analysis of **4** (PDF)

## #

Present Addresses University of Groningen, Faculty of Science and Engineering, Nijenborgh 3, 9747 AG Groningen, The Netherlands

## Abbreviations

[^35^S]­GTPγS: [^35^S] guanosine 5′-*O*-γ-thiotriphosphate)
CH_2_Br_2_: dibromomethane
CH_3_(CH_2_)_2_COOCO­(CH_2_)_2_CH_3_: butyryl anhydride
CH_3_(CH_2_)_9_COCl: undecanoyl chloride
CHS: cholesteryl hemisuccinate
CNS: central nervous system
CXCR2: CXC motif chemokine receptor 2
CXCR4: CXC motif chemokine receptor 4
DDM: dodecyl maltoside
DMSO: dimethyl sulfoxide
ESI: electrospray ionization
GCI: grating-coupled interferometry
GPCR: G protein-coupled receptor
HA-tag: human influenza hemagglutinin-tag
His-tag: histidine-tag
LTC4: leukotriene C4
LTD4: leukotriene D4
LTE4: leukotriene E4
MOE: molecular operating environment
MW: microwave
POCl_3_: phosphoryl oxychloride
SEM: standard error of the mean
SPR: surface plasmon resonance
TBAF·3H_2_O: tetrabutylammonium fluoride trihydrate
*t*-BuONO: *tert*-butylnitrite
TLC: thin layer chromatography
TMSBr: trimethylsilyl bromide
TMSCl: chlorotrimethylsilane
UDP: uridine diphosphate
UDP-g: UDP-glucose
MOE: molecular operating environment
PDB: protein data bank.
