# Supplementary material for: Structure-Guided Prioritization and Synthesis of New Ligands for GPR17 Receptor
Source: ACS Omega. 2026 Jun 6;11(24):35411–20. doi: 10.1021/acsomega.6c00828 (PMC13294945; doi:10.1021/acsomega.6c00828)
Supplement: Supplementary file 1 [file ao6c00828_si_001.pdf]

Structure-Guided Prioritization and Synthesis of New Ligands for GPR17 Receptor

Marco Rabuffetti[1,†], Francesca Rinaldi[2], Luca Palazzolo[3], Davide Bianchi[3], Maria Letizia Trincavelli[4], Irene Balloni[4], Simona Daniele[4], Stefano Capaldi[5], Giovanna Speranza[1], Ivano Eberini[3], Enrica Calleri[2,*]

[1] Dipartimento di Chimica, Università degli Studi di Milano, via Golgi 19, 20133 Milano, Italy

[2] Dipartimento di Scienze del Farmaco, Università di Pavia, via Taramelli 12, 27100 Pavia, Italy

[3] Dipartimento di Scienze Farmacologiche e Biomolecolari "Rodolfo Paoletti", Università degli Studi di Milano, via Giuseppe Balzaretti 9, 20133 Milano, Italy

[4] Dipartimento di Farmacia, Università di Pisa, via Bonanno 6, 56126 Pisa, Italy

[5] Dipartimento di Biotecnologie, Università di Verona, Strada Le Grazie 15, 37134 Verona, Italy

Supporting information

Table of contents

S1. Results of in silico computations S2
S2. Synthesis of inosinic and guanylic acids (1-4) and their intermediates S8
S3. HPLC data for the purification of compounds 1, 2, 3 and 10 .... S12
S4. ^{1}H, ^{13}C and ^{31}P and NMR spectra of compounds 1, 2, 3 and 4 .... S13
S5. Results of GCI kinetic analyses S21

S1. Results of in silico computations

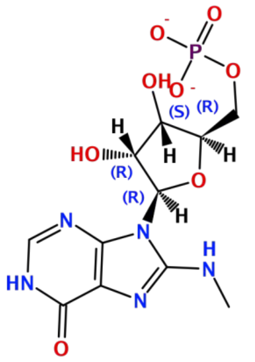

Compound 1

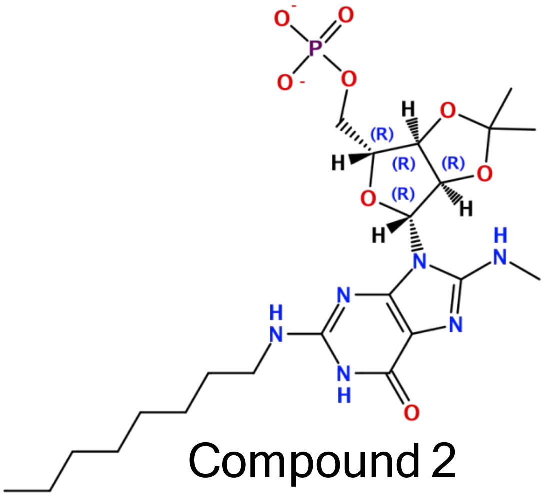

Compound 2

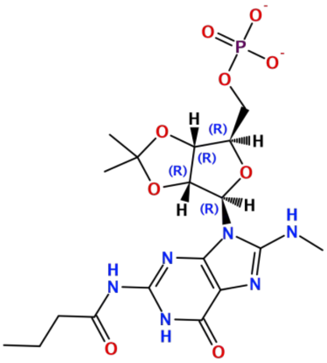

Compound 3

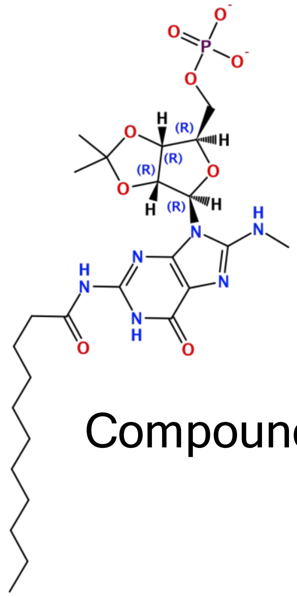

Compound 4

Figure S1. Top scoring chemical structures derived from *in silico* computations.

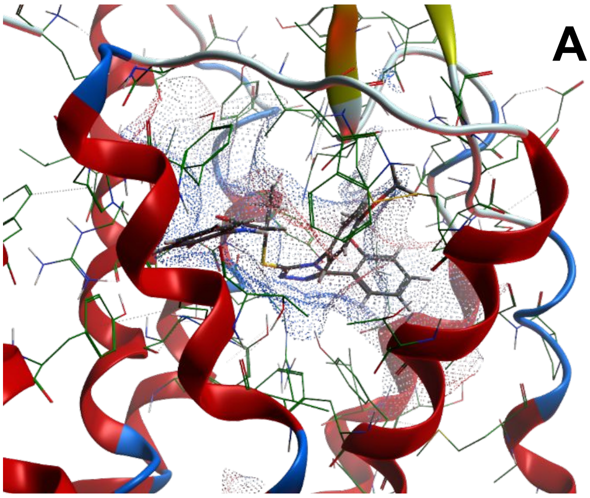

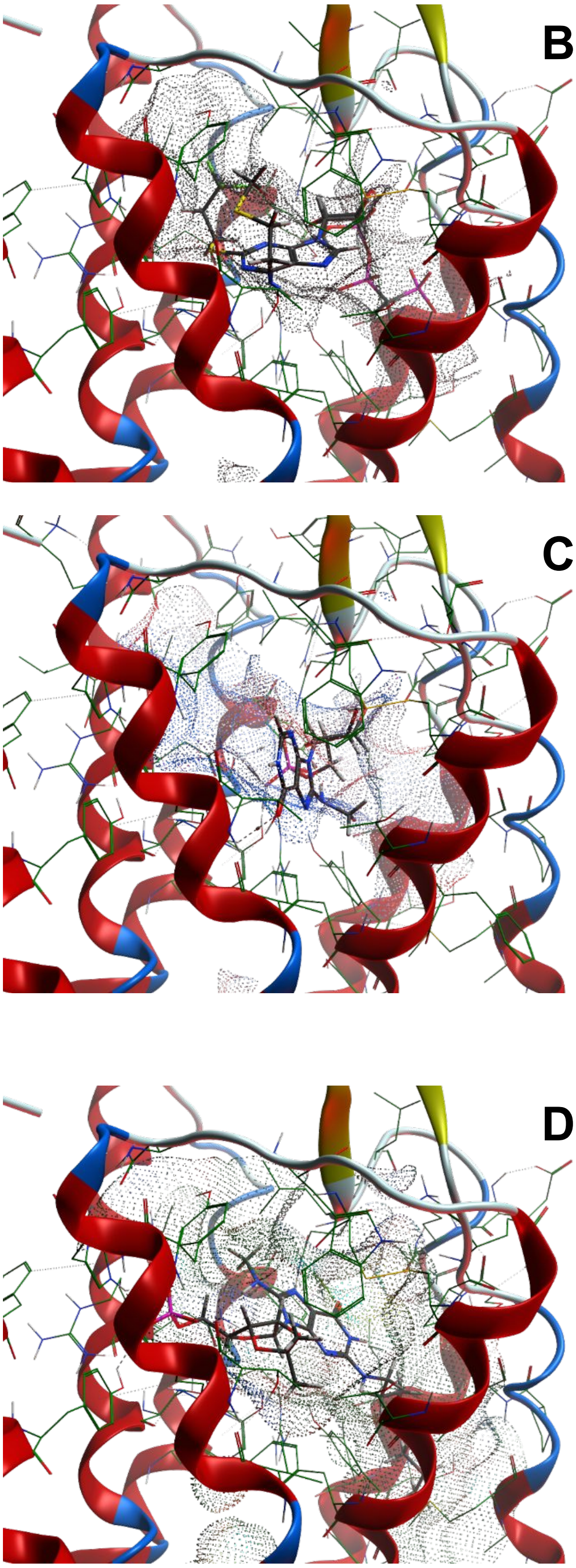
B
C
D

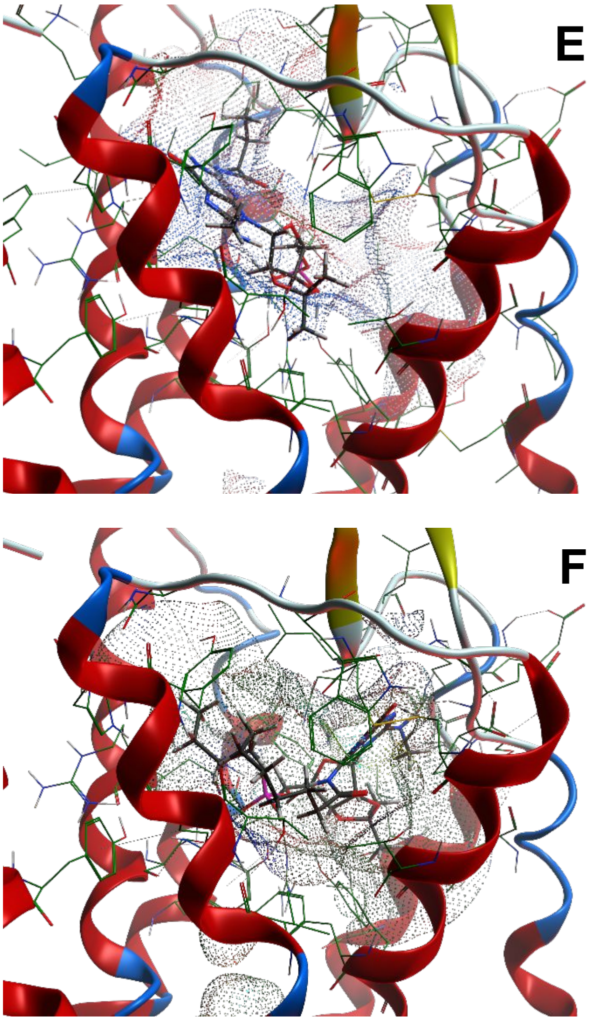

Figure S2. 3D Van der Waals surface representation of the ligand-receptor complexes for Asinex 1 (**A**), Cangrelor (**B**) and compounds **1** (**C**), **2** (**D**), **3** (**E**) and **4** (**F**).

Molecular models showing the top-scoring ligands in complex with the GPR17 binding pocket. The Van der Waals surface of the receptor is rendered in transparent grey, and ligands are shown in color-coded stick representation to highlight shape complementarity and occupancy of the binding site.

A

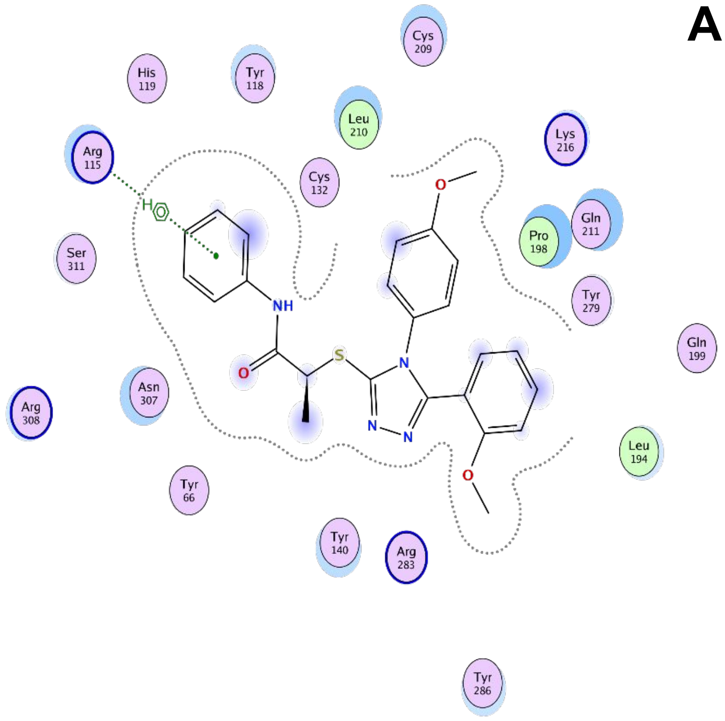

Cys 209
His 119
Tyr 118
Leu 210
Lys 216
Arg 115
Cys 132
O
Gln 211
Pro 198
Ser 311
NH
Tyr 279
S
N
Gln 199
O
Asn 307
Arg 308
N
N
Leu 194
O
Tyr 66
Tyr 140
Arg 283
Tyr 286

B

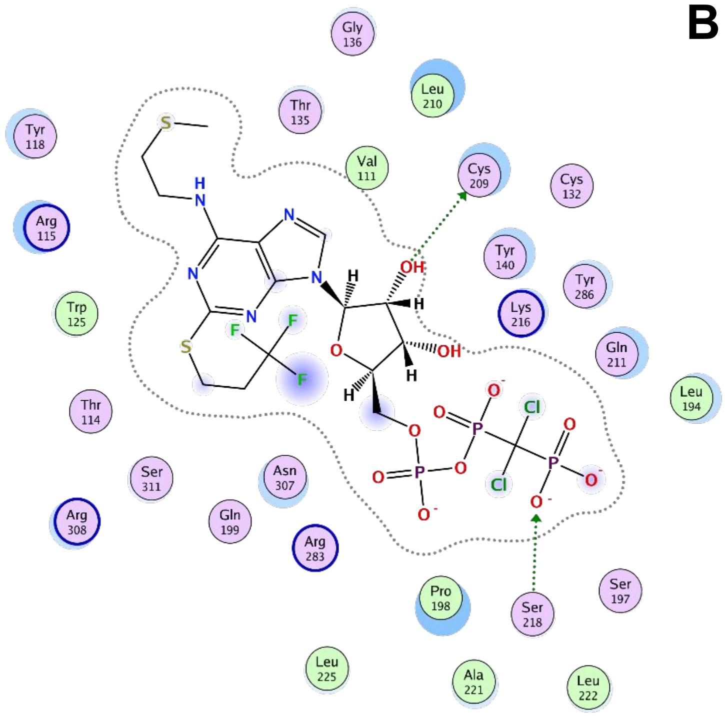

Gly 136
Leu 210
Thr 135
Tyr 118
S
Val 111
Cys 209
Cys 132
H
N
Arg 115
N
Tyr 140
N
OH
Tyr 286
N
H
H
Lys 216
Trp 125
N
F
F
O
OH
Gln 211
S
F
H
Leu 194
Thr 114
H
O
Cl
O
P
O
Cl
P
O
Ser 311
Asn 307
O
P
O
O
Cl
O
Arg 308
Gln 199
O
Arg 283
Pro 198
Ser 218
Ser 197
Leu 225
Ala 221
Leu 222

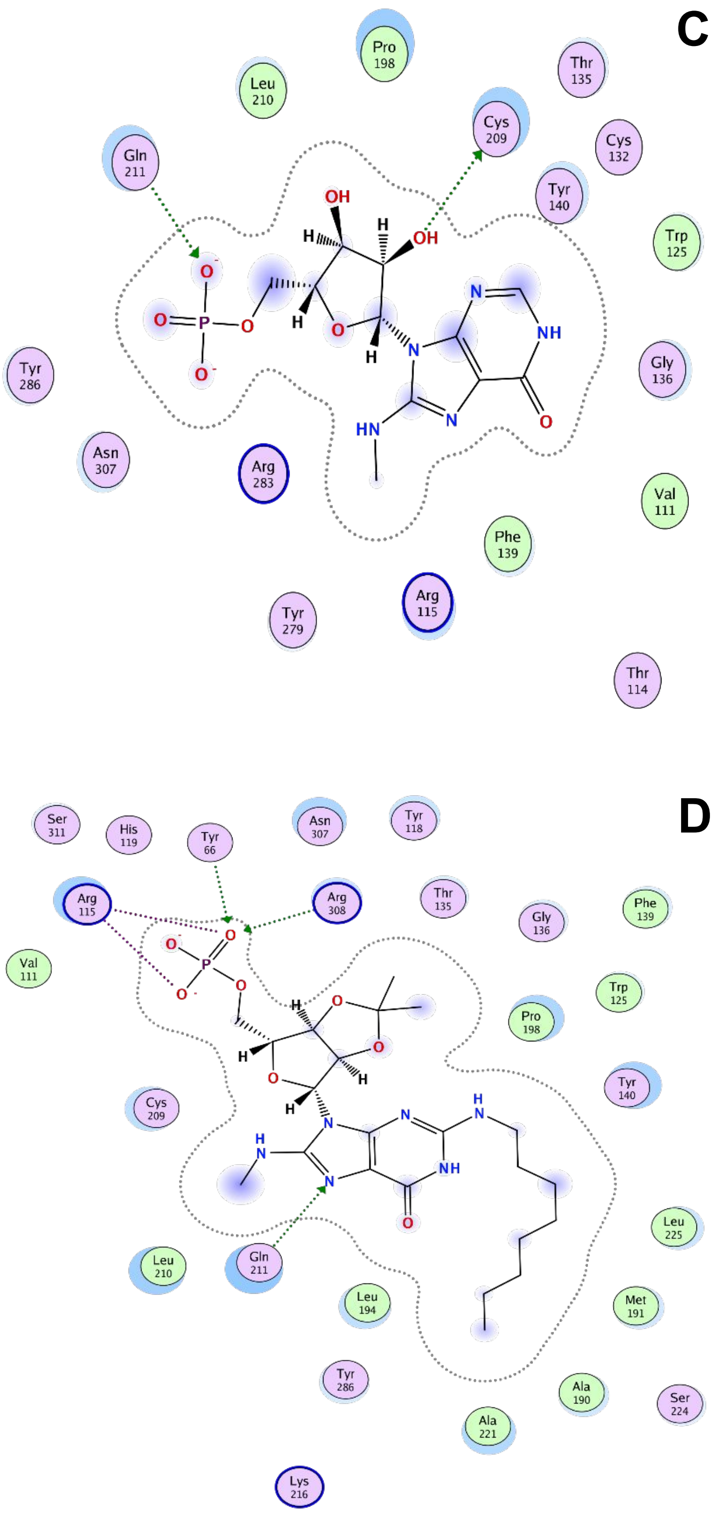
C
Pro 198
Thr 135
Leu 210
Cys 209
Cys 132
Gln 211
Tyr 140
Trp 125
Tyr 286
Gly 136
Asn 307
Arg 283
Val 111
Phe 139
Arg 115
Tyr 279
Thr 114
OH
H
OH
O
O
P
O
O
NH
N
N
N
HN
O
D
Ser 311
His 119
Tyr 66
Asn 307
Tyr 118
Arg 115
Arg 308
Thr 135
Gly 136
Phe 139
Val 111
Trp 125
Pro 198
Tyr 140
Cys 209
Leu 225
Leu 210
Gln 211
Leu 194
Met 191
Tyr 286
Ala 190
Ser 224
Ala 221
Lys 216

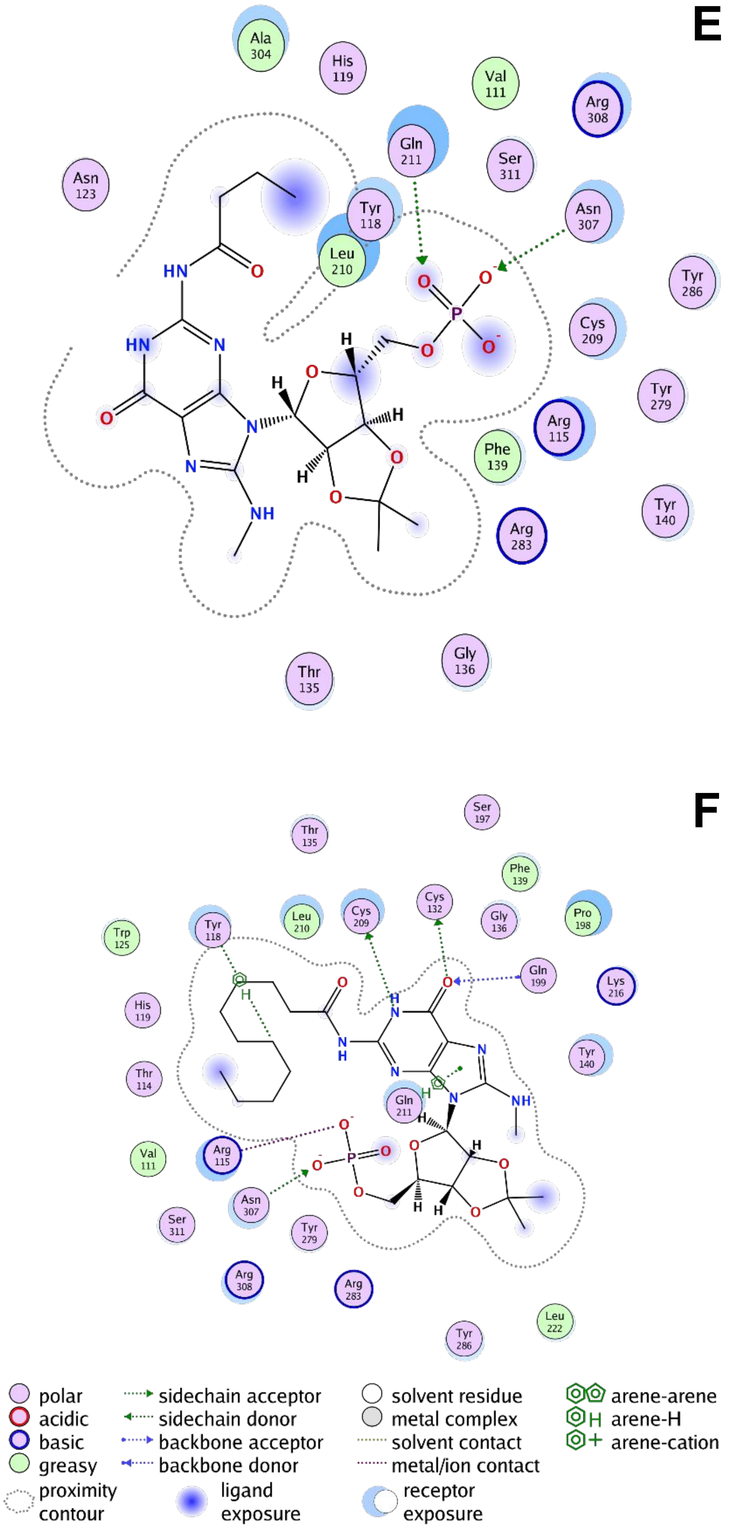

Figure S3. 2D interaction diagrams of ligand binding within the GPR17 binding site for Asinex 1 (**A**), Cangrelor (**B**) and compounds **1** (**C**), **2** (**D**), **3** (**E**) and **4** (**F**).

Two-dimensional interaction maps for the top-scoring ligands, highlighting key hydrogen bonds, hydrophobic contacts, and π–π interactions with GPR17 binding site residues. Diagrams were generated using molecular docking results to illustrate the molecular basis of ligand recognition.

S2. Synthesis of inosinic and guanylic acids (1-4) and their intermediates

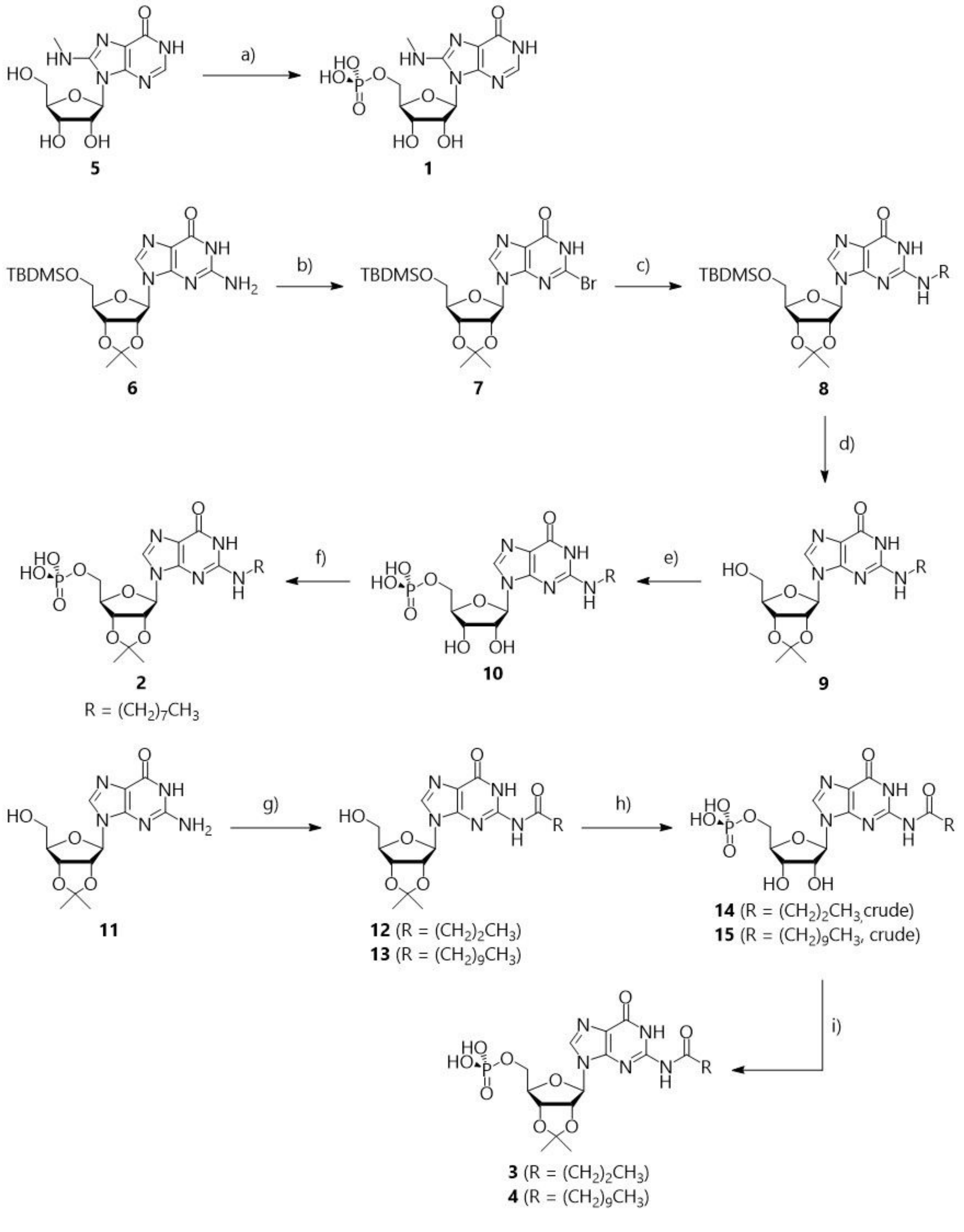

Scheme S1. Synthetic strategy for the preparation of target compounds **1**, **2**, **3** and **4**.
Reagents and conditions:

a), e) and h) i) triethylphosphate, 50 °C;
ii) $POCl_3$, H_2O, 0 °C;
iii) H_2O, 6 M NaOH (to pH 2);
iv) 70 °C

(76% for **1**, 73% for **10**, n.d. for **14** and **15**)

b) TMSBr, *t*-BuONO, CH_2Br_2, -10 °C → 5-10 °C (69%)
c) *n*-Octylamine, EtOH, MW, 110 °C, 300 W, 6 h (93%)
d) TBAF·3H_2O, THF (60%)
f) and i): 2,2-dimethoxypropane, *p*-toluenesulfonic acid monohydrate, acetone/DMF, reflux (81% for **2**, 30% for **3** (calculated from **12**) and 79% for **4** (calculated from **13**))
g) i) chlorotrimethylsilane, pyridine, CH_2Cl_2;
ii) RCOX (X = OCO$(CH_2)_2CH_3$ for **12** and Cl for **13**);
iii) 1 M HCl, THF
(63% for **12** and 62% for **13**)

8-Methylaminominoinosine (5). The title compound was synthesized as previously described.[1]

R**$_f$:** 0.18 (CH_2Cl_2-MeOH, 4:1)

^{1}H NMR (DMSO-*d*$_6$, 400 MHz): δ (ppm) 12.08 (br s, 1H), 7.82 (s, 1H), 6.81 (q, *J* = 4.5 Hz, 1H), 5.86 (d, *J* = 7.3 Hz, 1H), 5.68 (t, *J* = 4.6 Hz, 1H), 5.29 (d, *J* = 6.5 Hz, 1H), 5.13 (d, *J* = 4.3 Hz, 1H), 4.57 (dd, *J* = 12.6, 6.7 Hz, 1H), 4.13-4.08 (m, 1H), 3.96 (q, *J* = 2.3 Hz, 1H), 3.68-3.62 (m, 2H), 2.84 (d, *J* = 4.6 Hz, 3H).

^{13}C NMR (DMSO-*d*$_6$, 100 MHz): δ (ppm) 156.3, 152.2, 148.5, 143.1, 122.6, 87.4, 86.4, 71.7, 71.3, 62.2, 29.8.

MS (ESI$^+$): *m/z* calcd for $[C_{11}H_{15}N_5O_5]^+$: 297.11; found: 166.1 $[M\text{-ribosyl}+H]^+$, 188.1 $[M\text{-ribosyl}+Na]^+$, 298.1 $[M+H]^+$, 320.1 $[M+Na]^+$, 617.1 $[2M+Na]^+$.

2’,3’-*O*-Isopropylideneguanosine (11). The title compound was synthesized according to literature.[2]

R**$_f$:** 0.32 (CH_2Cl_2-MeOH, 9:1)

^{1}H NMR (DMSO-*d*$_6$, 400 MHz): δ (ppm) 10.55 (br s, 1H), 7.92 (s, 1H), 6.52 (br s, 2H), 5.93 (d, *J* = 2.8 Hz, 1H), 5.19 (dd, *J* = 6.3, 2.8 Hz, 1H), 5.11 (t, *J* = 5.4 Hz, 1H), 4.97 (dd, *J* = 6.3, 3.0 Hz, 1H), 4.12 (td, *J* = 5.1, 3.2 Hz, 1H), 3.56 (dd, *J* = 11.2, 4.8 Hz, 1H), 3.51 (dd, *J* = 11.6, 5.2 Hz, 1H), 1.51 (s, 3H), 1.31 (s, 3H).

^{13}C NMR (DMSO-*d*$_6$, 400 MHz): δ (ppm) 157.2, 154.2, 151.2, 136.3, 117.2, 113.5, 88.9, 87.1, 84.0, 81.6, 62.1, 27.5, 25.7.

MS (ESI$^+$): *m/z* calcd for $[C_{13}H_{17}N_5O_5]^+$: 323.12; found: 345.9 $[M+Na]^+$, 669.0 $[2M+Na]^+$.

General procedure for the synthesis of *N*^{2}-acyl-2’,3’-*O*-isopropylideneguanosines (12 and 13)

Chlorotrimethylsilane (2.70 mL, 21.27 mmol, 7.09 equiv) was added dropwise to a suspension of **11** (969 mg, 3.00 mmol, 1.00 equiv) in a dry CH_2Cl_2/pyridine mixture (5:1 v/v, 90 mL) under inert atmosphere at 0 °C. After stirring the resulting mixture at room temperature for 4 h, the system was cooled to 0 °C and the appropriate acylating agent (3.30 mmol, 1.10 equiv) was added dropwise over 5 minutes. The reaction was stirred at room temperature and monitored by TLC (CH_2Cl_2 saturated with NH_3-MeOH, 9:1) until complete consumption of the starting material. The solution was washed with 1 M HCl (2 x 60 mL), a saturated $NaHCO_3$ solution (2 x 60 mL) and brine (1 x 40 mL), then dried over Na_2SO_4. The solvent was evaporated under reduced pressure, removing residual pyridine by co-evaporation with toluene. The resulting yellow foam was dissolved in a 1 M HCl/THF mixture (1:1 v/v, 30 mL) and stirred at room temperature for 2 h. CH_2Cl_2 (150 mL) was added, the solution was washed with H_2O (90 mL) and dried over Na_2SO_4. After removing the solvent under reduced pressure, the resulting crude was purified by flash column chromatography (5% MeOH in CH_2Cl_2).

N^2-Butyryl-2',3'-*O*-isopropylideneguanosine (12). The title product was obtained as a white powder (740 mg, 1.88 mmol, 63%).

***Rf*:** 0.29 (CH_2Cl_2-MeOH, 20:1)

^{1}H NMR ($CDCl_3$, 400 MHz): δ (ppm) 12.28 (br s, 1H), 9.82 (br s, 1H), 8.14 (s, 1H), 5.93 (d, *J* = 3.0 Hz, 1H), 5.19 (dd, *J* = 6.0, 3.0 Hz, 1H), 5.15 (dd, *J* = 6.2, 1.7 Hz, 1H), 4.39 (q, *J* = 2.7 Hz, 1H), 4.38-4.24 (br s, 1H), 4.00 (dd, *J* = 12.2, 3.0 Hz, 1H), 3.82 (dd, *J* = 12.2, 2.6 Hz, 1H), 2.57 (t, *J* = 7.4 Hz, 2H), 1.76 (hex, *J* = 7.4 Hz, 2H), 1.60 (s, 3H), 1.37 (s, 3H), 1.01 (t, *J* = 7.4 Hz).

^{13}C NMR ($CDCl_3$, 100 MHz): δ (ppm) 175.2, 155.1, 147.6, 147.2, 138.4, 121.3, 114.0, 91.8), 86.3, 83.7, 81.0, 62.3, 38.7, 27.1, 25.0, 18.0, 13.3.

MS (ESI$^+$): *m/z* calcd for $[C_{17}H_{23}N_5O_6]^+$: 393.16; found: 394.52 $[M+H]^+$, 416.71 $[M+Na]^+$, 787.38 $[2M+H]^+$, 809.46 $[2M+Na]^+$.

N^2-*n*-Undecanoyl-2',3'-*O*-isopropylideneguanosine (13). The title product was obtained as a light-yellow powder (914 mg, 1.86 mmol, 62%).

R_f: 0.37 (CH_2Cl_2-MeOH, 20:1)

^{1}H NMR ($CDCl_3$, 400 MHz): δ (ppm) 12.27 (br s, 1H), 9.81 (br s, 1H), 8.07 (s, 1H), 5.91 (d, *J* = 3.2 Hz, 1H), 5.19 (dd, *J* = 6.0, 3.3 Hz, 1H), 5.09 (dd, *J* = 6.0, 1.8 Hz, 1H), 4.39 (br dd, *J* = 5.1, 3.0 Hz, 1H), 3.96 (dd, *J* = 12.0, 2.6 Hz, 1H), 3.80 (dd, *J* = 12.1, 2.6 Hz, 1H), 2.56 (t, *J* = 7.5 Hz, 2H), 1.76-1.64 (m, 2H), 1.59 (s, 3H), 1.40-1.21 (m, 17H), 0.89 (t, *J* = 6.8 Hz, 3H).

^{13}C NMR ($CDCl_3$, 100 MHz): δ (ppm) 174.9, 154.0, 147.1, 146.4, 139.0, 119.6, 113.2, 91.2, 85.9, 83.0, 80.4, 61.4, 36.1, 30.9, 28.5, 28.4, 28.3, 28.2, 28.0, 26.2, 24.2, 23.7, 21.6, 13.1.

MS (ESI$^+$): *m/z* calcd for $[C_{24}H_{37}N_5O_6]^+$: 491.27; found: 514.59 $[M+Na]^+$, 1005.38 $[2M+Na]^+$.

2',3'-*O*-Isopropylidene-5'-*O*-*t*-butyldimethylsilylguanosine (6). The title compound was synthesized according to literature.[3]

R_f: 0.54 (CH_2Cl_2-MeOH, 9:1)

^{1}H NMR ($CDCl_3$, 400 MHz): δ (ppm) 12.05 (br s, 1H), 7.79 (s, 1H), 6.29 (br s, 2H), 6.02 (br d, *J* = 2.3 Hz, 1H), 5.19 (br dd, *J* = 6.0, 2.0 Hz, 1H), 4.94 (dd, *J* = 6.2, 2.8 Hz, 1H), 4.36 (dd, *J* = 7.0, 4.0 Hz, 1H), 3.87 (dd, *J* = 11.2, 4.0 Hz, 1H), 3.80 (dd, *J* = 11.3, 4.0 Hz, 1H), 1.64 (s, 3H), 1.43 (s, 3H), 0.90 (s, 9H), 0.073 (s, 3H), 0.068 (s, 3H).

^{13}C NMR ($CDCl_3$, 100 MHz): δ (ppm) 159.0, 153.6, 151.3, 136.4, 117.4, 114.1, 90.3, 87.0, 84.7, 81.2, 63.5, 27.3, 25.5, 25.9, 18.4, -5.4, -5.5.

MS (ESI$^+$): *m/z* calcd for $[C_{19}H_{31}N_5O_5Si]^+$: 437.21; found: 438.1 $[M+H]^+$, 460.2 $[M+Na]^+$, 897.2 $[2M+Na]^+$.

2-Bromo-2',3'-*O*-isopropylidene-5'-*O*-*t*-butyldimethylsilylinosine (7). The title compound was synthesized from **6** (69%) as previously described.[4]

R_f: 0.56 (CH_2Cl_2-MeOH, 9:1)

^{1}H NMR ($CDCl_3$, 400 MHz): δ (ppm) 12.85 (br s, 1H), 8.10 (s, 1H), 6.15 (d, *J* = 2.8 Hz, 1H), 5.06 (dd, *J* = 6.1, 2.6 Hz, 1H), 4.95 (dd, *J* = 6.1, 2.7 Hz, 1H), 4.41 (q, *J* = 3.4 Hz, 1H), 3.92 (dd, *J* = 11.4, 3.4 Hz, 1H), 3.83 (dd, *J* = 11.3, 3.7 Hz, 1H), 1.66 (s, 3H), 1.42 (s, 3H), 0.91 (s, 9H), 0.11 (s, 3H), 0.10 (s, 3H).

^{13}C NMR ($CDCl_3$, 100 MHz): δ (ppm) 158.9, 148.3, 138.8, 133.1, 123.8, 114.4, 90.7, 87.1, 85.2, 81.1, 63.5, 27.3, 25.4, 25.9, 18.4, -5.4, -5.5.

MS (ESI$^+$): *m/z* calcd for $[C_{19}H_{29}{}^{79}BrN_4O_5Si]^+$: 500.11; found: 523.0 $[(^{79}Br)M+Na]^+$, 525.1 $[(^{81}Br)M+Na]^+$, 1023.0 $[2(^{79}Br)M+Na]^+$, 1025.0 $[(^{79}Br)M+(^{81}Br)M+Na]^+$, 1027.0 $[2(^{81}Br)M+Na]^+$.

***N*^{2}-*n*-Octyl-2',3'-*O*-isopropylidene-5'-*O*-*t*-butyldimethylsilylguanosine (8).** *n*-Octylamine (0.16 mL, 0.97 mmol, 3.03 equiv) was added to a solution of **7** (160 mg, 0.32 mmol, 1.00 equiv) in dry EtOH (3.20 mL) under inert atmosphere. The resulting mixture was stirred at 110 °C under MW irradiation (300 W) for 6 h. The solvent was removed under reduced pressure and the obtained orange oil was purified by flash column chromatography (3% MeOH in CH_2Cl_2) to get **8** as a light-yellow powder (164 mg, 0.30 mmol, 93%).

***R_f*:** 0.59 (CH_2Cl_2-MeOH, 9:1)

^1H NMR ($CDCl_3$, 400 MHz): δ (ppm) 11.81 (br s, 1H), 7.71 (br s, 1H), 6.93 (br s, 1H), 5.94 (br s, 1H), 5.18 (br d, J = 4.3 Hz, 1H), 4.84 (br dd, J = 5.2, 2.1 Hz, 1H), 4.27 (br dd, J = 6.8, 4.2 Hz, 1H), 3.78 (br dd, J = 11.1, 3.5 Hz, 1H), 3.70 (dd, J = 11.0, 4.6 Hz, 1H), 3.32 (br t, J = 5.4 Hz, 2H), 1.64-1.50 (m, 5H), 1.36-1.05 (m, 13H), 0.86-0.71 (m, 12H), -0.04 (s, 3H), -0.05 (s, 3H).

^{13}C NMR ($CDCl_3$, 100 MHz): δ (ppm) 158.8, 153.1, 151.0, 136.1, 116.8, 113.8, 91.0, 87.4, 84.4, 81.7, 63.7, 41.7, 31.9, 31.9, 29.5, 29.4, 27.2, 25.4, 25.6, 25.8, 22.6, 18.3, 14.1, -5.43, -5.46.

MS (ESI$^+$): *m/z* calcd for $[C_{27}H_{47}N_5O_5Si]^+$: 549.33; found: 550.2 $[M+H]^+$, 572.3 $[M+Na]^+$, 1121.4 $[2M+Na]^+$.

***N*^{2}-*n*-Octyl-2',3'-*O*-isopropylideneguanosine (9).** TBAF·$3H_2O$ (51 mg, 0.16 mmol, 1.23 equiv) was added to a solution of **8** (74 mg, 0.13 mmol, 1.00 equiv) in THF (1.50 mL) under inert atmosphere. The resulting yellow mixture was stirred at room temperature for 6 h, during which time a precipitate was formed. The suspension was diluted with CH_2Cl_2 (3 mL), washed with H_2O (2 x 2 mL) and brine (1 x 2 mL) and dried over Na_2SO_4. The solvent was removed under reduced pressure and the resulting yellow oil was purified by flash column chromatography (8% MeOH in CH_2Cl_2) to obtain **9** as an off-white powder (34 mg, 0.08 mmol, 60%).

***R_f*:** 0.35 (CH_2Cl_2-MeOH, 9:1)

^1H NMR (DMSO-*d_6*, 400 MHz): δ (ppm) 10.62 (br s, 1H), 7.88 (s, 1H), 6.53 (t, J = 5.4 Hz, 1H), 5.99 (d, J = 2.8 Hz, 1H), 5.31 (dd, J = 6.0, 2.8 Hz, 1H), 4.98 (t, J = 5.6 Hz, 1H), 4.91 (dd, J = 6.2, 3.1 Hz, 1H), 4.12 (dt, J = 5.3, 2.8 Hz, 1H), 3.47-3.57 (m, 2H), 3.31-3.21 (m, 2H), 1.64-1.50 (m, 2H), 1.53 (s, 3H), 1.32 (s, 3H), 1.24-1.29 (m, 10H), 0.85 (t, J = 6.8 Hz, 3H.

^{13}C NMR (DMSO-*d_6*, 100 MHz): δ (ppm) 157.6, 153.5, 151.1, 137.2, 117.8, 113.8, 89.6, 87.4, 84.2, 82.2, 62.5, 41.4, 32.1, 29.6, 29.5, 29.4, 27.9, 26.0, 27.2, 22.9, 14.7.

MS (ESI$^+$): *m/z* calcd for $[C_{21}H_{33}N_5O_5]^+$: 435.25; found: 436.08 $[M+H]^+$, 458.30 $[M+Na]^+$, 871.21 $[2M]^+$, 893.15 $[2M+Na]^+$.

S3. HPLC data for the purification of compounds 1, 2, 3 and 10

**Compound**	**Elution program** Time (min)	% A	% B	t_R
**1**	0	100	0	14.9
	30	70	30	
	30.5	100	0	
	35	100	0	
**2**	0	95	5	42.8
	5	95	5	
	50	40	60	
	50.5	95	5	
	55	95	5	
**3**	0	95	5	51.6
	5	95	5	
	55	0	100	
	55.5	95	5	
	60	95	5	
**10**	0	100	0	17.3
	5	100	0	
	35	60	40	
	35.5	100	0	
	40	100	0	

Table S1. HPLC data for the purification of compounds **1, 2, 3** and **10**

S4. ¹H, ¹³C and ³¹P and NMR spectra of compounds 1, 2, 3 and 4

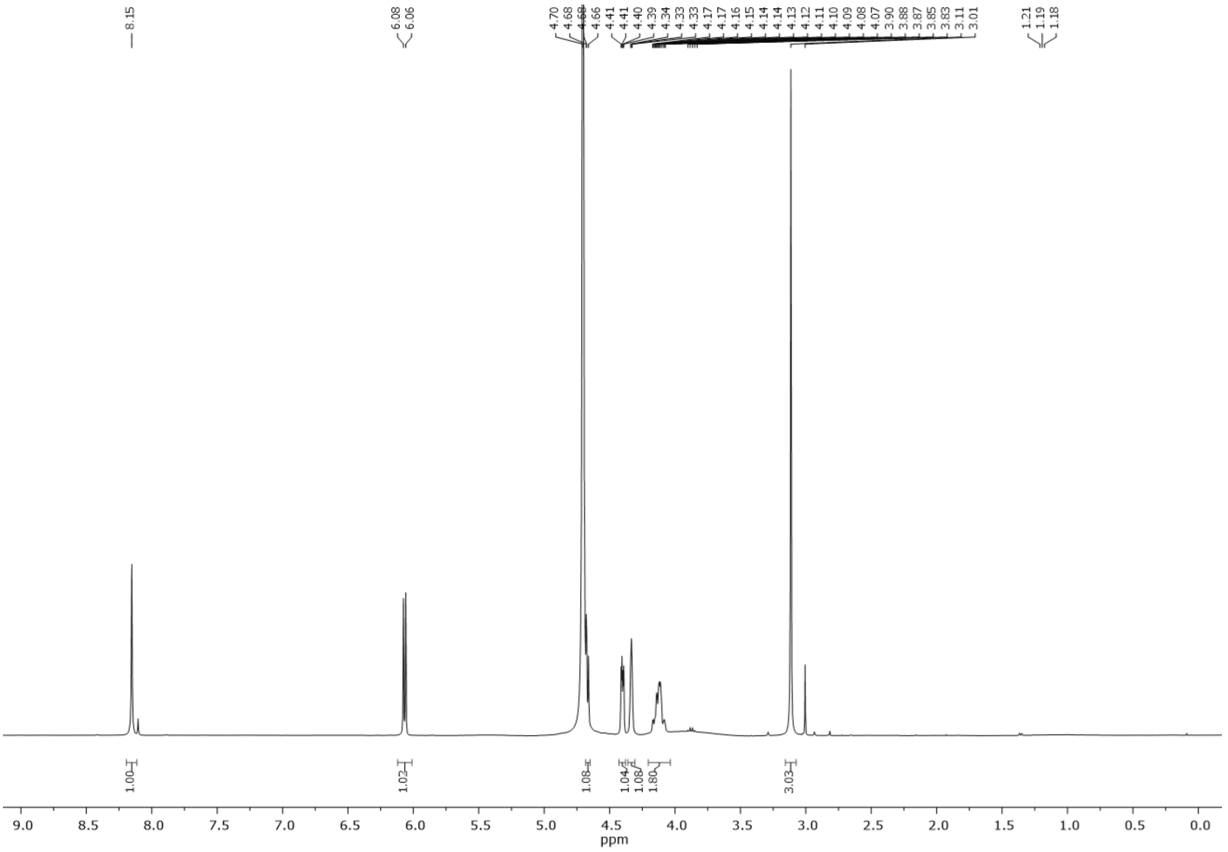

Figure S4. ^{1}H NMR spectra of 8-methylaminoinosinic acid (**1**).

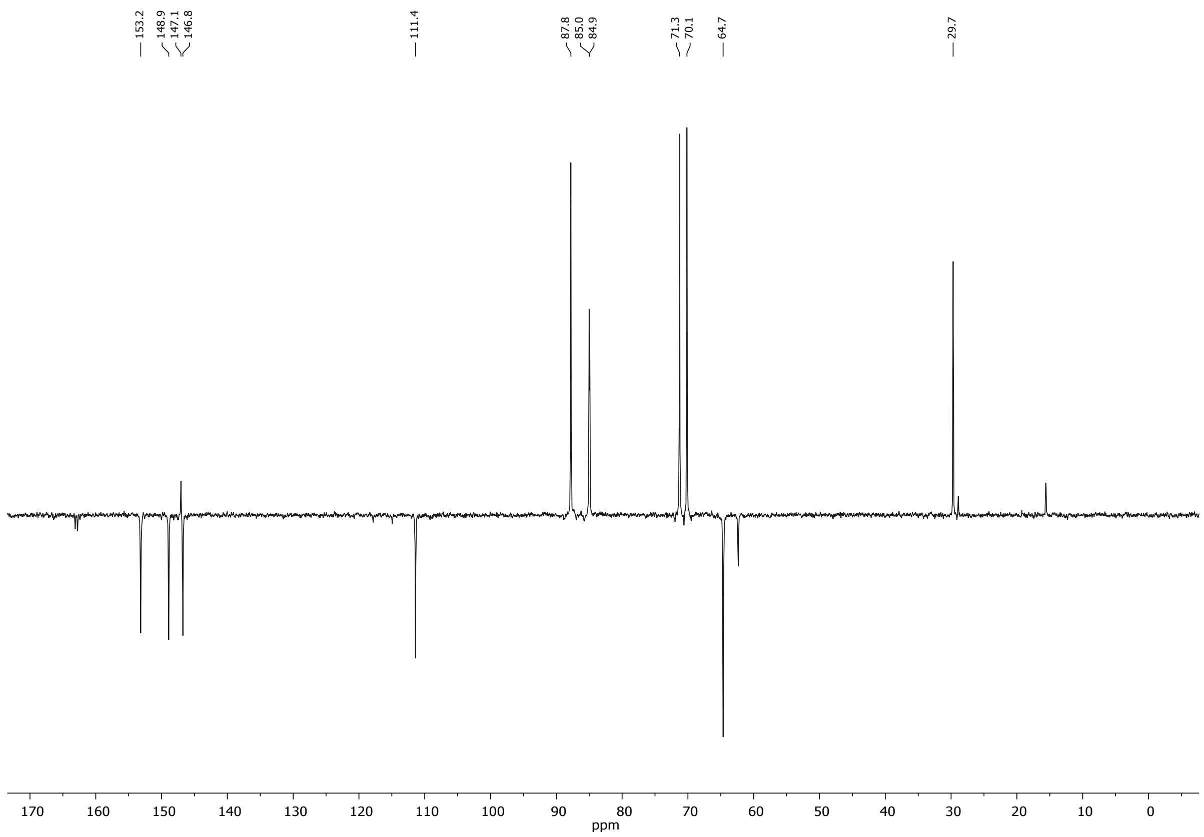

Figure S5. ^{13}C NMR spectra of 8-methylaminoinosinic acid (**1**).

Figure S6. ^{31}P NMR spectra of 8-methylaminoinosinic acid (**1**).

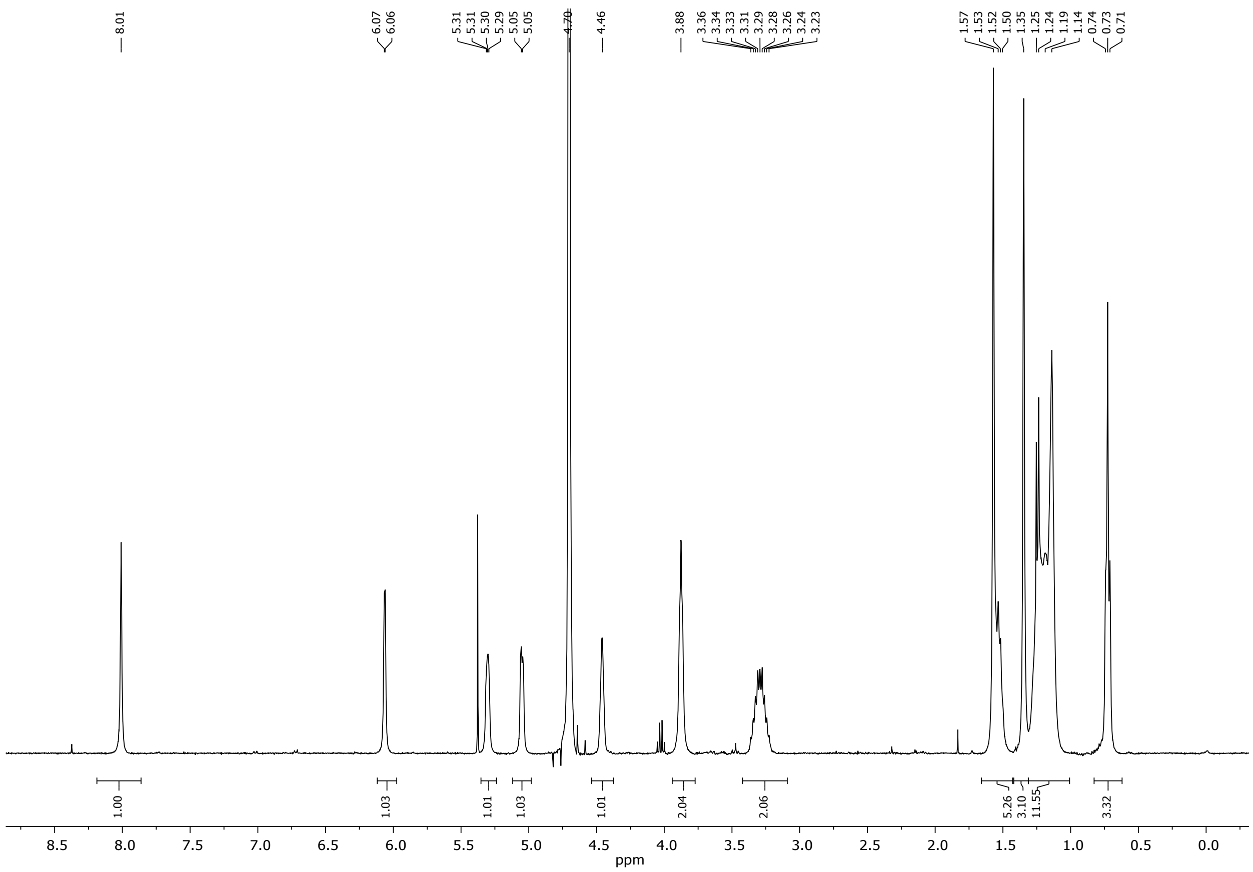

Figure S7. ^{1}H NMR spectra of N^2-*n*-octyl-2’,3’-*O*-isopropilydeneguanylic acid (**2**).

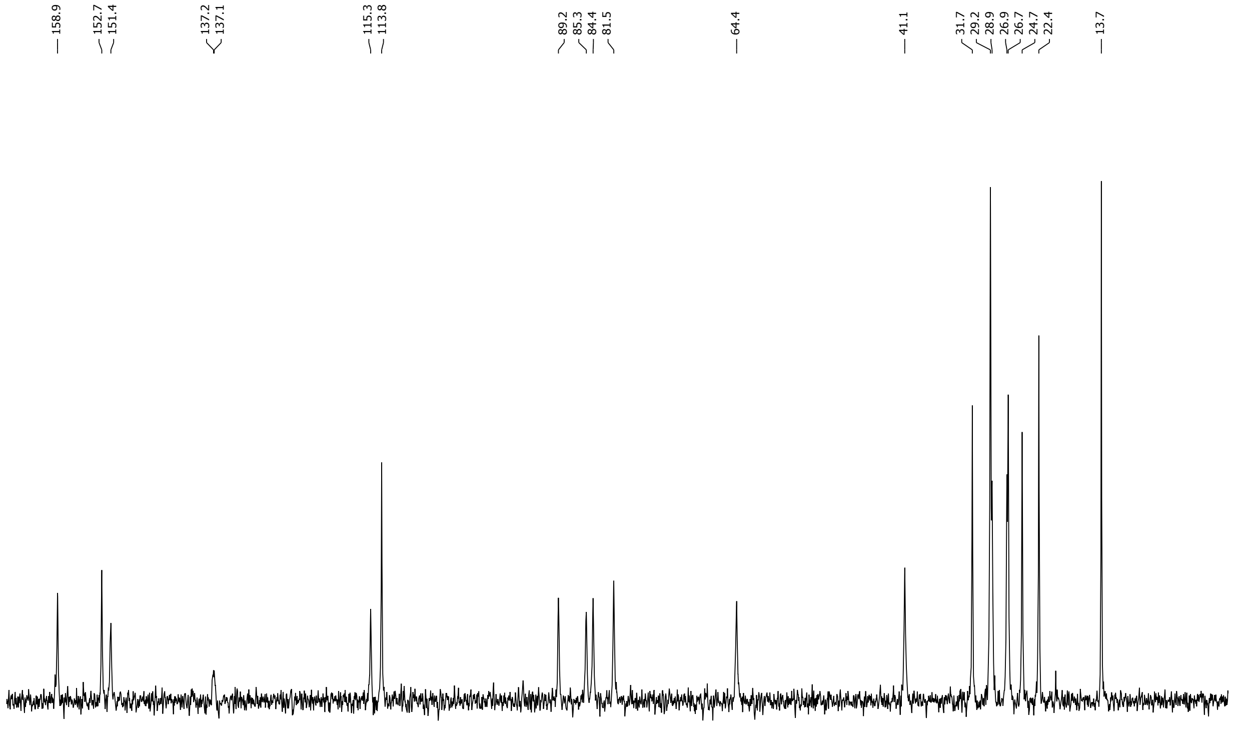

Figure S8. ^{13}C NMR spectra of N^2-*n*-octyl-2’,3’-*O*-isopropilydeneguanylic acid (**2**).

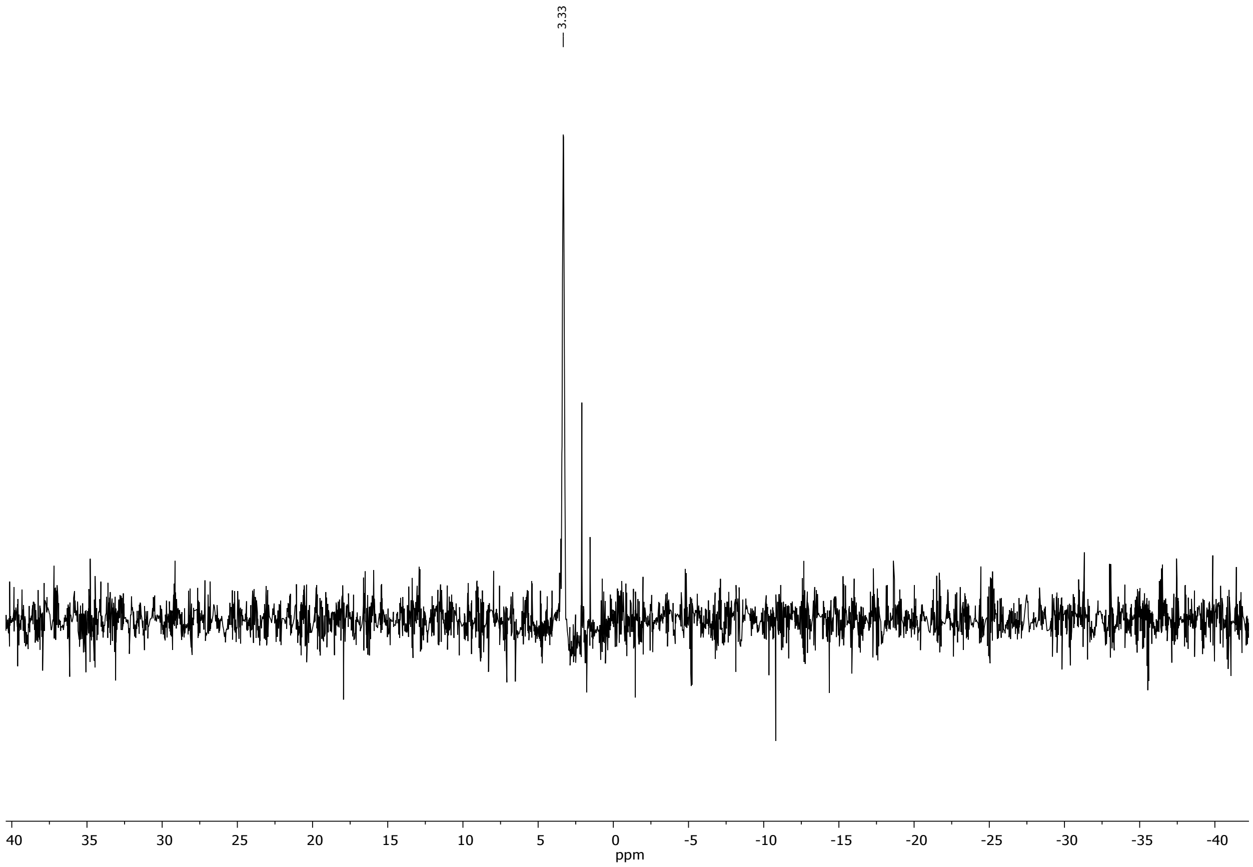

Figure S9. ^{31}P NMR spectra of N^2-*n*-octyl-2’,3’-*O*-isopropilydeneguanylic acid (**2**).

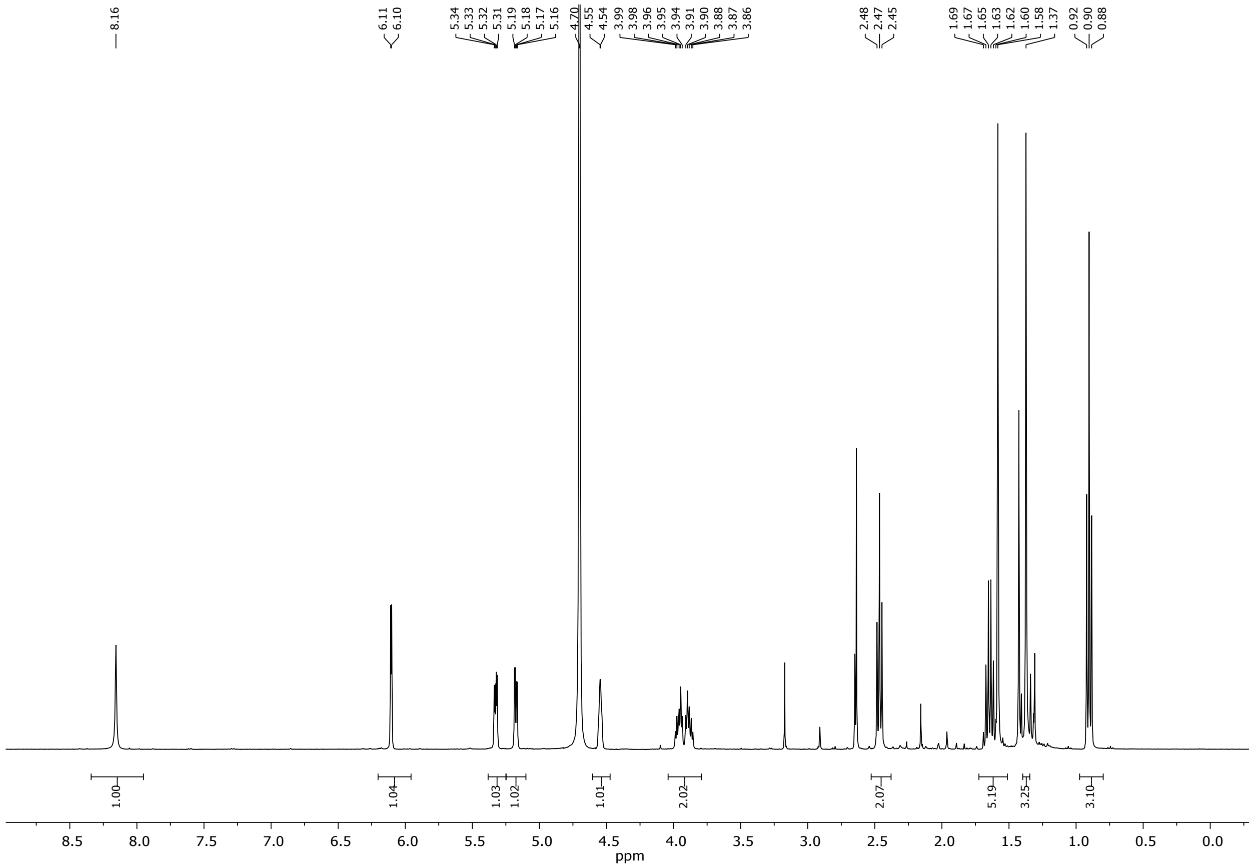

Figure S10. ^{1}H NMR spectra of *N*^{2}-*n*-butyryl-2',3'-*O*-isopropilydeneguanylic acid (**3**).

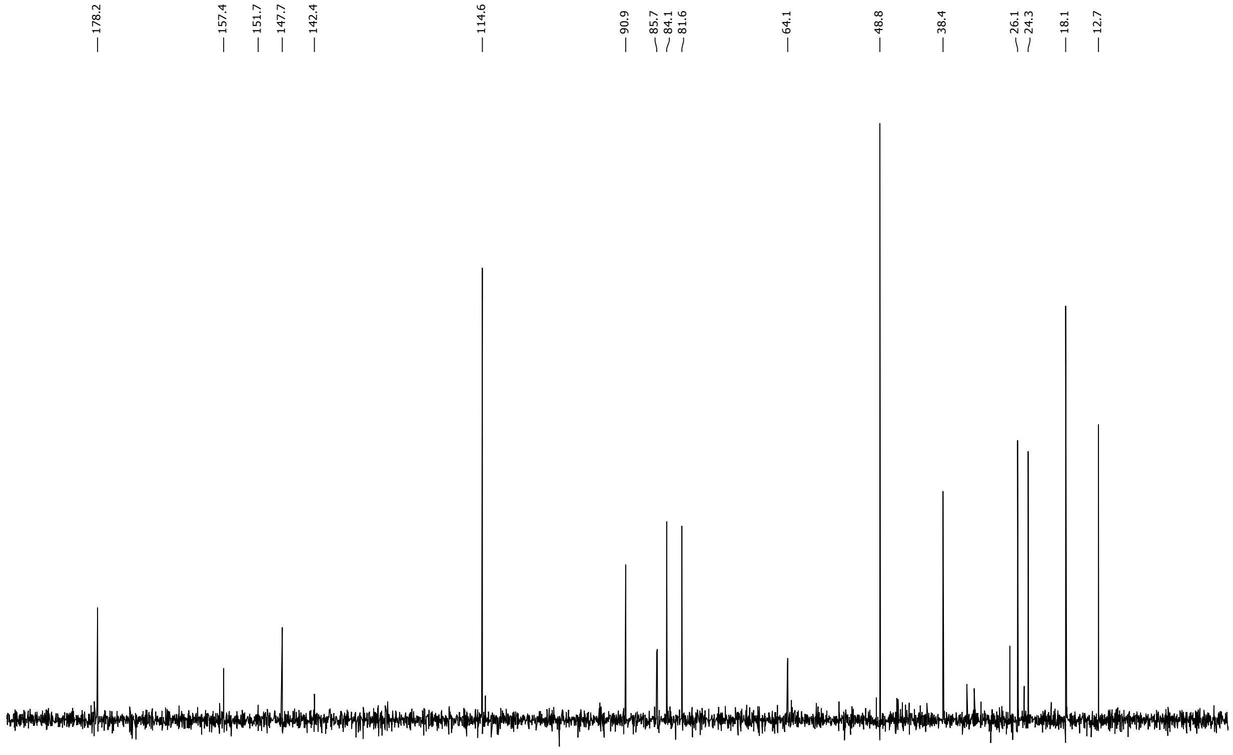

Figure S11. ^{13}C NMR spectra of *N*^{2}-*n*-butyryl-2',3'-*O*-isopropilydeneguanylic acid (**3**).

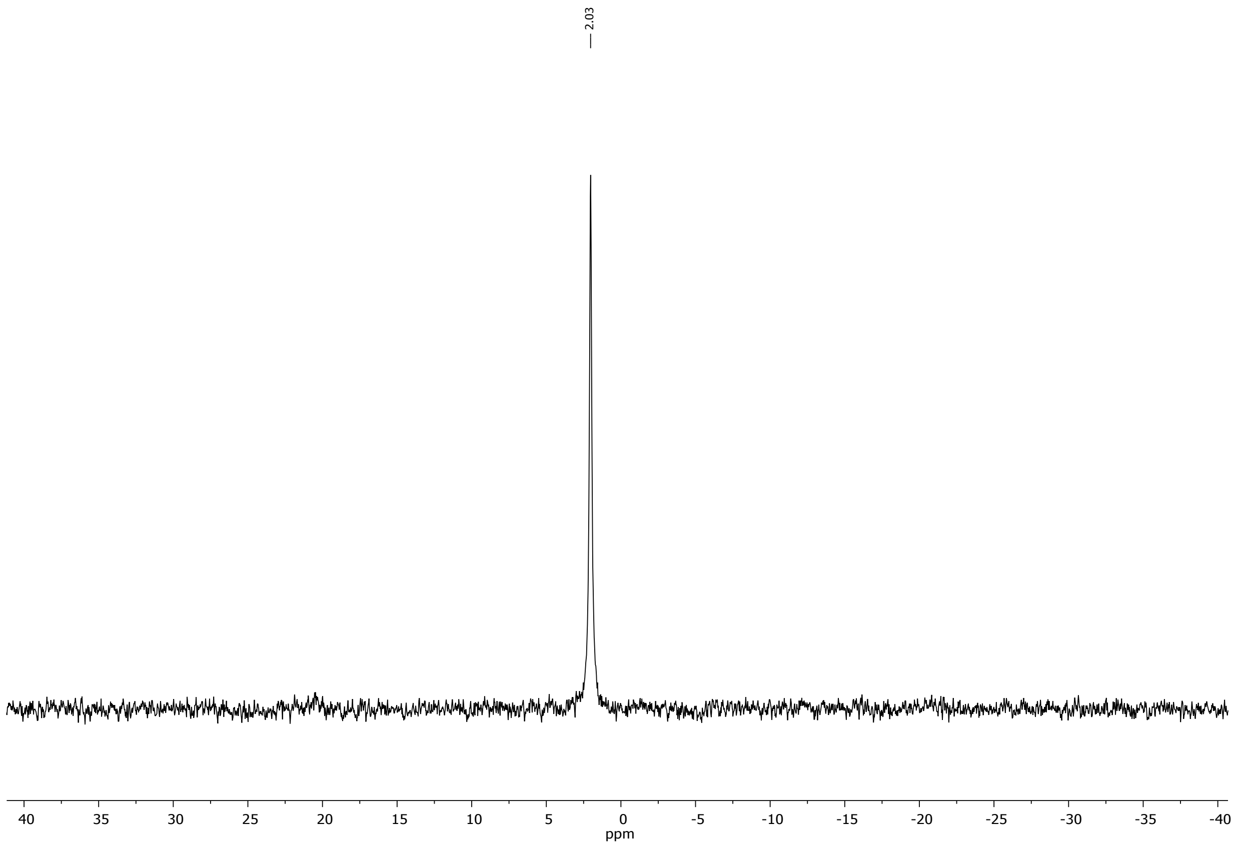

Figure S12. ^{31}P NMR spectra of N^2-*n*-butyryl-2',3'-*O*-isopropilydeneguanylic acid (**3**).

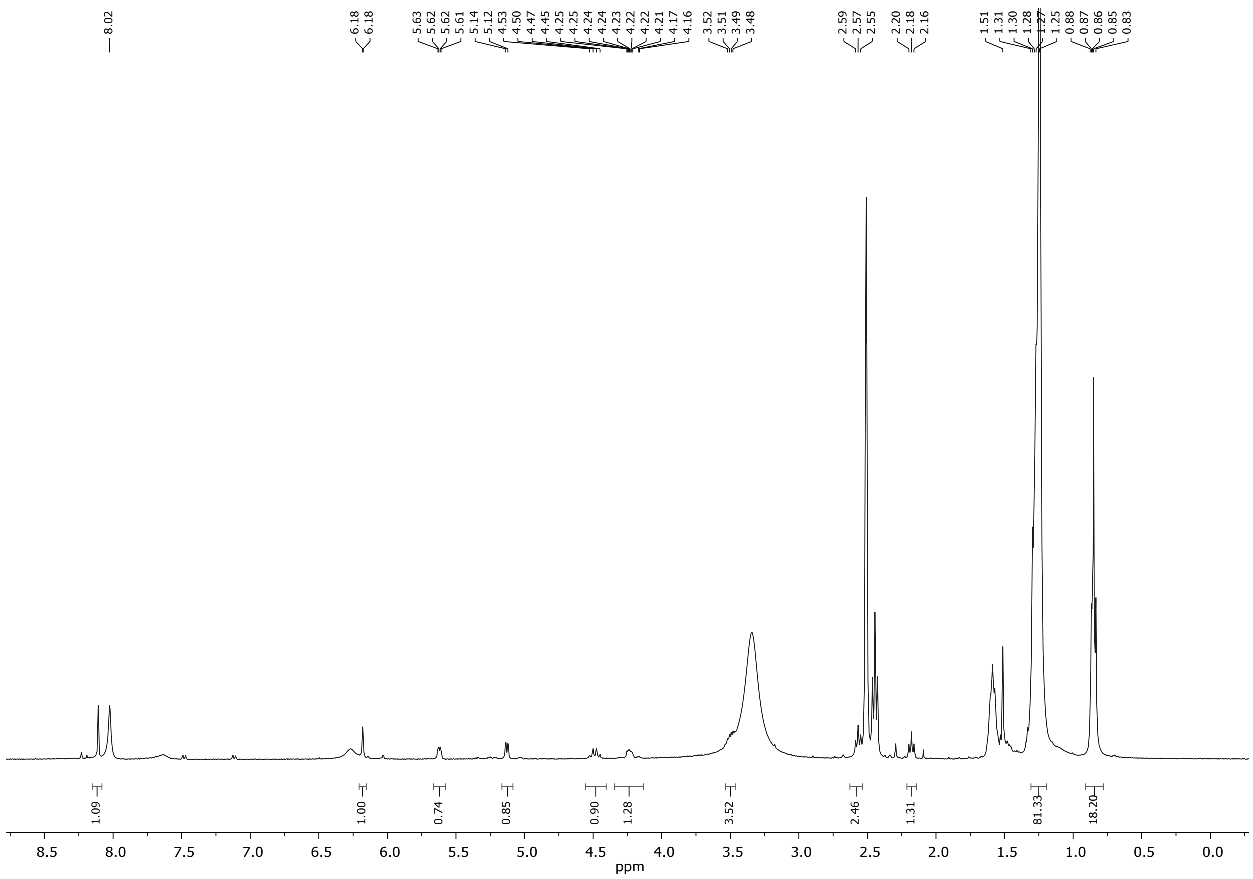

Figure S13. ^{1}H NMR spectra of N^2-*n*-undecanoyl-2',3'-*O*-isopropilydeneguanylic acid (**4**).

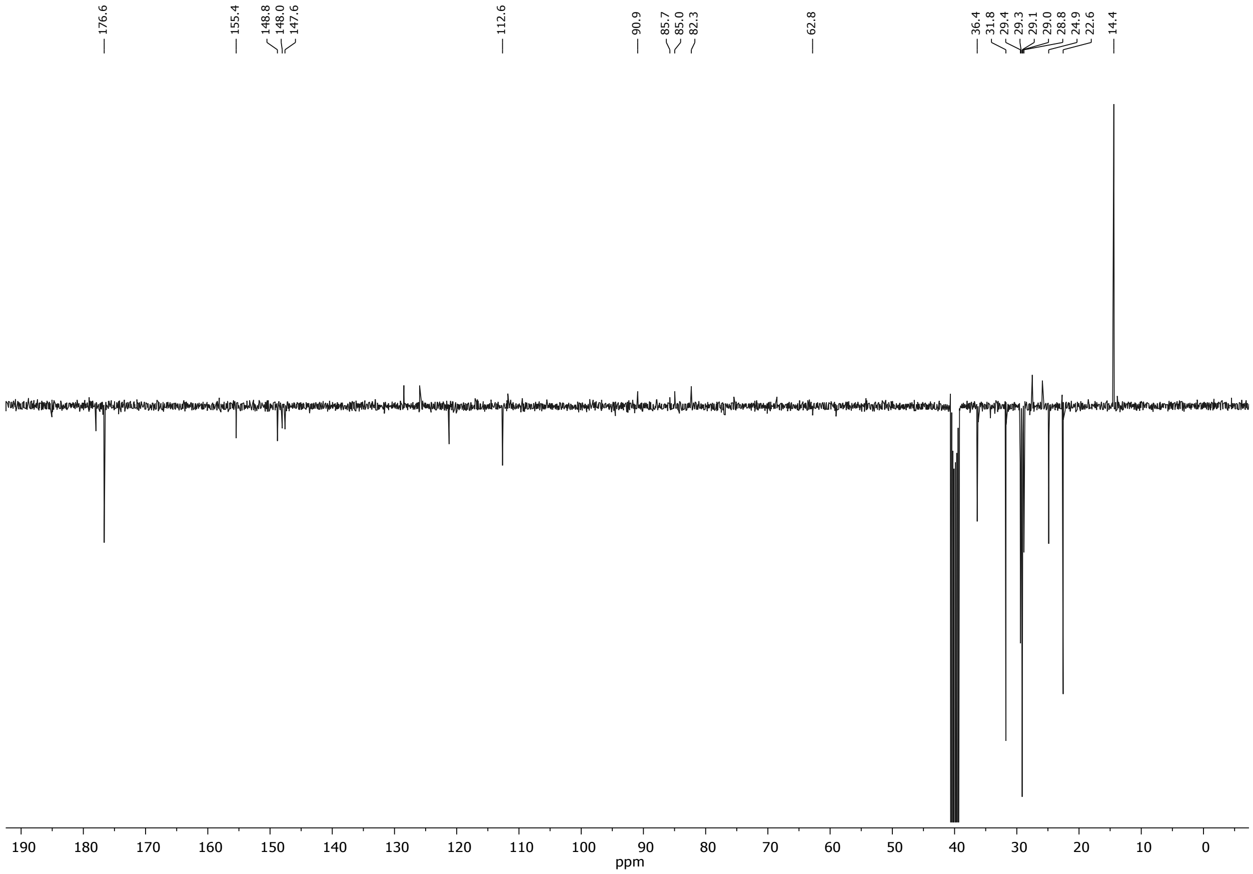

Figure S14. ^{13}C NMR spectra of N^2-*n*-undecanoyl-2',3'-*O*-isopropilydeneguanylic acid (**4**).

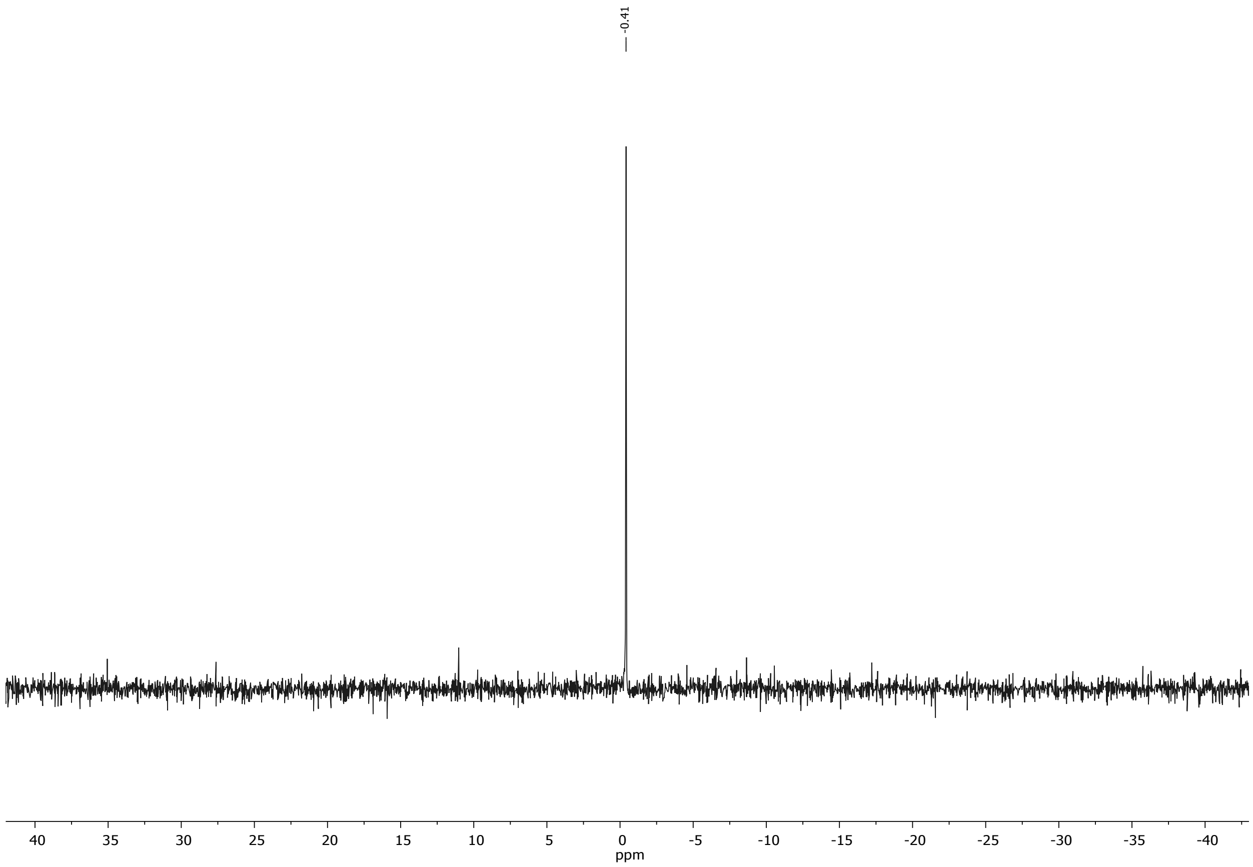

Figure S15. ^{31}P NMR spectra of *N*^{2}-*n*-undecanoyl-2',3'-*O*-isopropilydeneguanylic acid (**4**).

S5. Results of GCI kinetic analyses

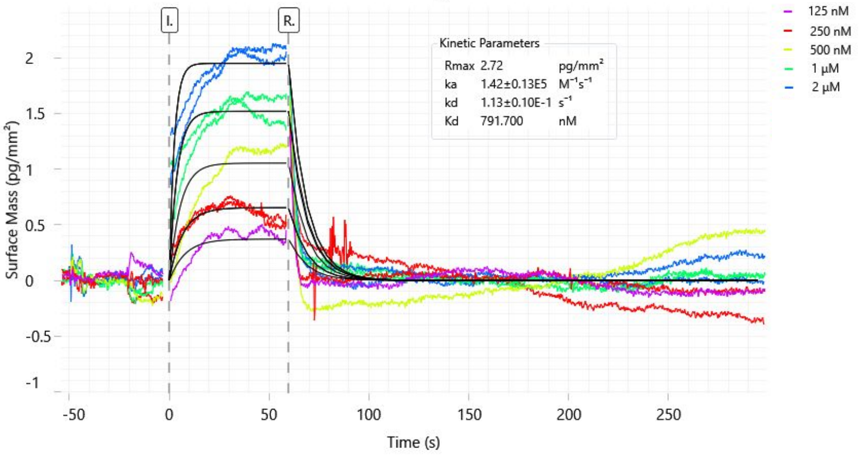

Figure S16. Sensorgrams deriving from the GCI kinetic analysis of Cangrelor.

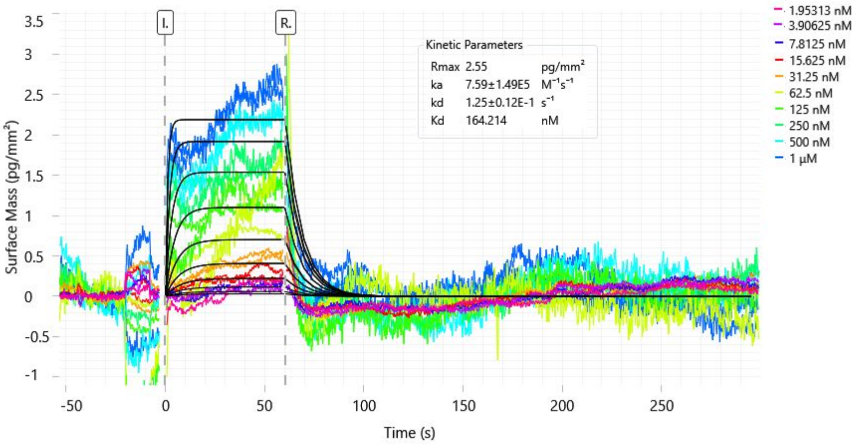

Figure S17. Sensorgrams deriving from the GCI kinetic analysis of Asinex 1.

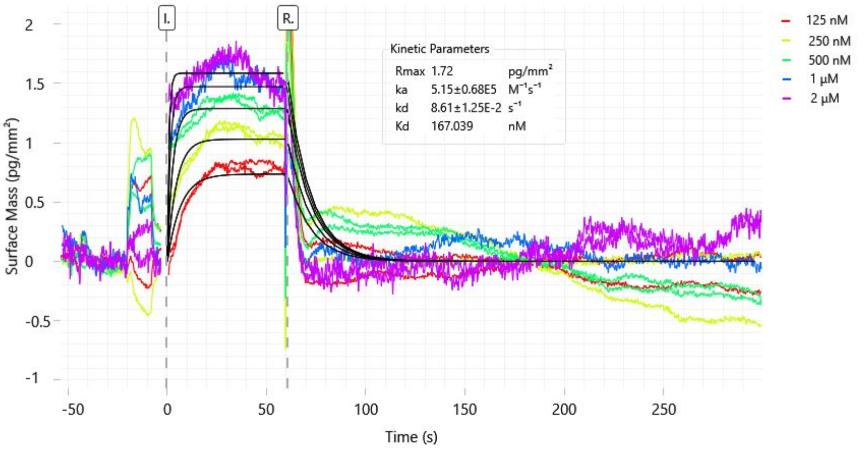

Figure S18. Sensorgrams deriving from the GCI kinetic analysis of **1**.

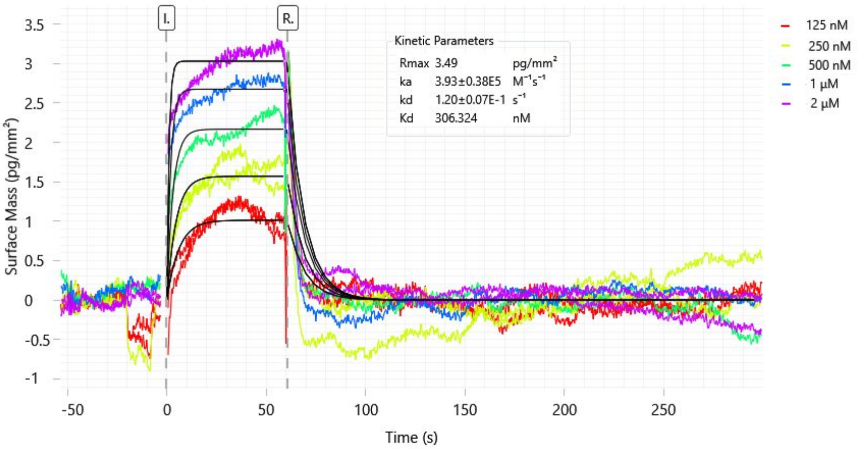

Figure S19. Sensorgrams deriving from the GCI kinetic analysis of **2**.

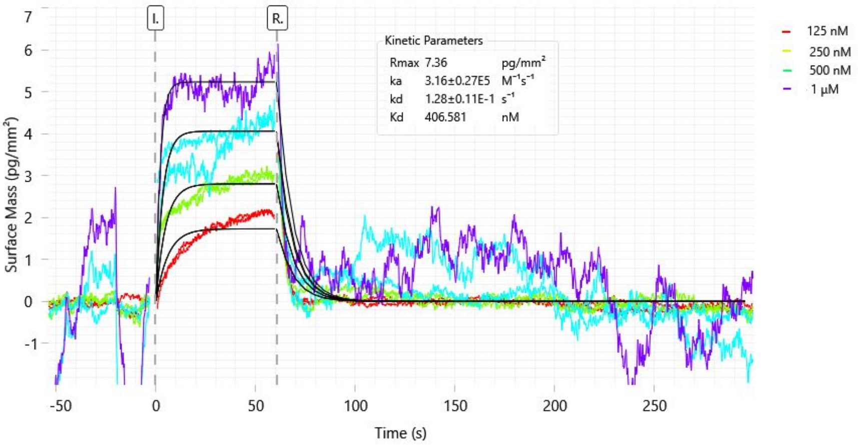

Figure S20. Sensorgrams deriving from the GCI kinetic analysis of **3**.

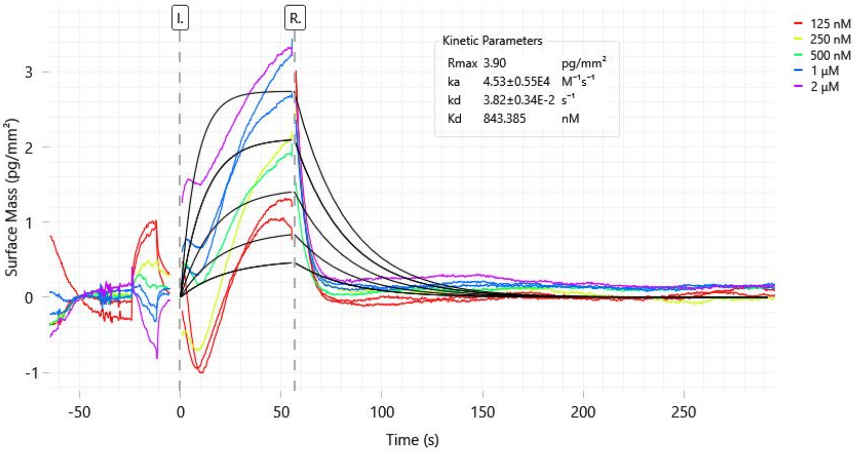

Figure S21. Sensorgrams deriving from the GCI kinetic analysis of **4**.

References

[1] M. Rabuffetti, G. Speranza, F. Rinaldi, D. Ubiali, G. Massolini, E. Calleri, A. Lo Bianco M. C. de Moraes, L. C. Rodrigues Pereira da Silva, A. Lavecchia *ChemMedChem* **2021**, *16*, 1325-1334.

[2] S. M. Devine, L. T. May, P.J. Scammells *Med. Chem. Commun.*, **2014**, *5*, 192-196.

[3] J. T. Davis, M. S. Kaucher, F. W. Kotch, M. A. Iezzi, B. C. Clover, K. M. Mullaugh *Org. Lett.* **2004**, *6*, 4265-4268.

[4] C. F. Morelli, V. Pappalardo, A. Brockhoff, S. Pieraccini, M. Sironi, S. Sangiorgio, L. Scarabattoli, G. Speranza, M. Rabuffetti *ChemistrySelect*. **2022**, *7*, e202204123.
